# Establishment of a integrative multi-omics expression database CKDdb in the context of chronic kidney disease (CKD)

**DOI:** 10.1038/srep40367

**Published:** 2017-01-12

**Authors:** Marco Fernandes, Holger Husi

**Affiliations:** 1 Institute of Cardiovascular and Medical Sciences, University of Glasgow, BHF Glasgow Cardiovascular Research Centre, 126 University Place, Glasgow, G12 8TA, UK

## Abstract

Complex human traits such as chronic kidney disease (CKD) are a major health and financial burden in modern societies. Currently, the description of the CKD onset and progression at the molecular level is still not fully understood. Meanwhile, the prolific use of high-throughput omic technologies in disease biomarker discovery studies yielded a vast amount of disjointed data that cannot be easily collated. Therefore, we aimed to develop a molecule-centric database featuring CKD-related experiments from available literature publications. We established the Chronic Kidney Disease database CKDdb, an integrated and clustered information resource that covers multi-omic studies (microRNAs, genomics, peptidomics, proteomics and metabolomics) of CKD and related disorders by performing literature data mining and manual curation. The CKDdb database contains differential expression data from 49395 molecule entries (redundant), of which 16885 are unique molecules (non-redundant) from 377 manually curated studies of 230 publications. This database was intentionally built to allow disease pathway analysis through a systems approach in order to yield biological meaning by integrating all existing information and therefore has the potential to unravel and gain an in-depth understanding of the key molecular events that modulate CKD pathogenesis.

Complex human disorders or traits such as chronic kidney disease (CKD) are nowadays a major health and financial burden in modern societies. Taking in account the last two decades the number of CKD-related deaths has risen by 82.3%, which is the third largest increase among the top 25 leading causes of death worldwide^1^. Moreover, the health costs associated with CKD morbidity as the treatment of end stage renal diseases (ESRD) in many developed countries can easily ascend to 3% of their internal health-care^2^.

The current CKD classification system comprehends five stages of decline of kidney function that is routinely assessed in the clinic through the measurement of the glomerular filtration rate (GFR) and also by the content of proteins found in urine and circulatory fluids; consequently this biochemical parameter can also be correlated with the degree of kidney damage^3^. Accordingly with the National Kidney Foundation, responsible for the establishment of the clinical assessment guidelines of Kidney Disease Outcomes Quality Initiative (NKF KDOQI), the current CKD classification system comprehends five stages of decline of kidney function (stage 1: GFR >90 mL/min/1.73 m2, stage 2: GFR 60–89 mL/min/1.73 m2, stage 3: GFR 30–59 mL/min/1.73 m2, stage 4: GFR 15–29 mL/min/1.73 m2, stage 5: GFR <15 mL/min/1.73 m2)^3^. Although, serum creatinine levels are being used as a biochemical parameter for GFR estimation and ultimately classification of the CKD stage, when it is used as a single parameter for diagnosis, it underestimates the presence of CKD at stage 3 or greater among older adults^4^. Therefore, we should reinforce the importance of finding novel biomarkers that could allow the distinction between CKD stages and in this way enabling an earlier assistance to patients.

During the past decade, major advances in the field of ‘omics technologies have led to an exponential growth in available experimental data. Most of the omics platforms provide high-throughput, detailed exploration of the genome, transcriptome, proteome and metabolome, through a variety of techniques including mRNA and miRNA arrays, next generation sequencing and mass spectrometry^5^.

The intrinsic nature of ‘omic data being noisy and usually on a very large-scale causes additional problems during analysis and concomitantly gives rise to other layers of complexity that are added due to the biological context, which is often highly diverse. For these reasons, establishment of ‘omics databases is particularly relevant in order to provide standardization and structured storage of information that in this way facilitates further data analysis, cross comparison and integration on a greater scale^6^.

Finding value in complex biological data streams, such as in high-throughput ‘omic platforms seems to be the upcoming era for the use of a combinatorial integrative approach through systems biology and bioinformatics resources. Thus, the latter approach has the potential to promote an unbiased and knowledge-driven platform for hypotheses generation in the context of disease modelling. As a consequence, traditional approaches using hypothesis-driven investigations in the study of complex human disorders or traits such as CKD are converging to a hypothesis-free systems approach to exploit the potential to yield an unbiased and novel testable hypothesis as an end-result.

One of the main gaps of the current “panorama” is the disparity between the fast generation of large-scale data and their in-depth analysis. Hence, a high volume of ‘omic data has been generated with the goal of discovery of novel disease biomarkers and although it found its way into publicly available databases, no effort has been made to combine and consolidate this resources in order to offer an integrative view of all studies.

Publicly available databases containing differential expression of miRNAs, genes, proteins and metabolites measured in a defined disease or in multi-diseases are still scarce with some rare exceptions as the case of general scope transcriptomics repositories such as NCBI Gene Expression Omnibus (GEO)^7^ and EBI Expression Atlas^8^. However, the core source for publicly available ‘omic data still resides in peer-reviewed publications, therefore we aimed to develop a curated molecule-centric database with multi-omics studies retrieved from the literature that encompasses differential expression data in CKD and other related traits that can lead to this disease with the ultimate goal to build a molecular disease model through a system approach. Thus, a global molecular chronic kidney disease database (CKDdb) was generated with data extracted from the literature. Manual curation was done to ensure the highest quality of all retrieved data.

## Results

### Database structure, deployment and navigation

**Figure 1:**
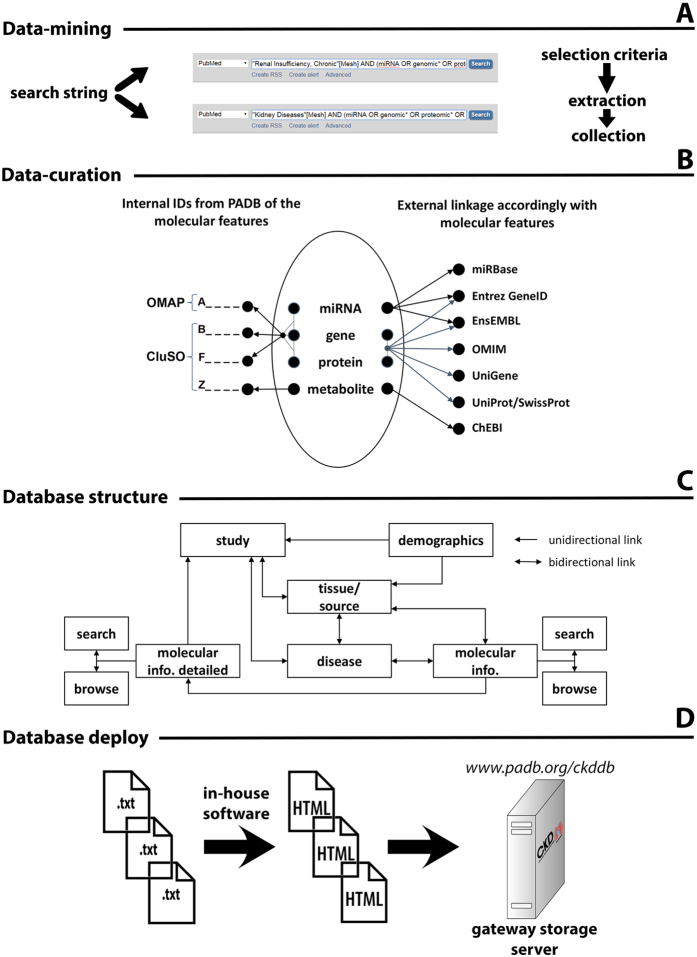
Stepwise description of the essential elements and main methodology used in the development of the database: from data-mining (**A**) till deployment (**D**). CKDdb database logo published under an Open Access license to Nature Publishing Group, a division of Macmillan Publishers Ltd.

**Figure 2:**
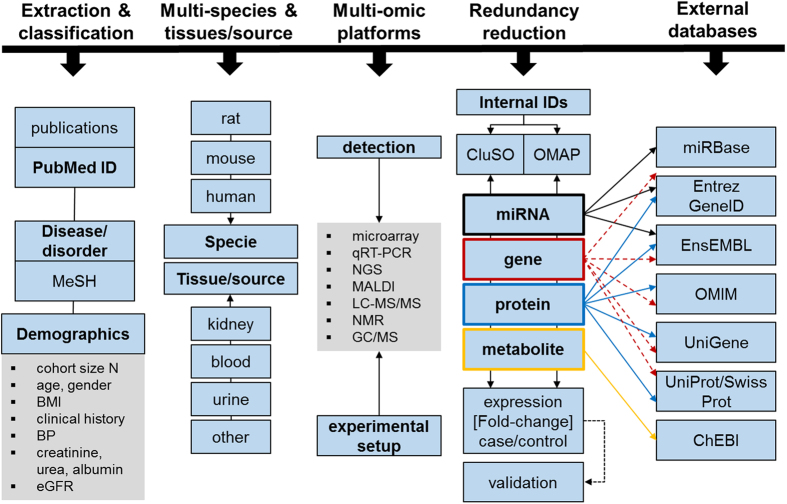
General scheme overview of the database structure and organisation.

The CKDdb database (Figures S1–S5) is based on pre-assembled and interlinked html files following a NoSQL database format, and all data is initially sourced from spreadsheets. There are 5 different spreadsheets relating to demographics (Figure S5), experimental setup, statistically found significant molecules, extended molecular information, and peptidomics data (peak profiling data). All of these tables follow the standard database nomenclature and structure, i.e. they are all linked to each other and to external databases (Figs 1 and 2) such as UniProt/SwissProt^9^, Chemical Entities of Biological Interest (ChEBI)^10^, NCBI gene^11^, miRbase^12^, Ensembl^13^ and Unigene^11^. Thus, in this way we emulated a database system by pre-assembling query outputs as e.g. grouping based on disease/tissue/molecule/etc.

For a matter of simplicity and usability, all collected data was stored in spreadsheets (but avoiding and solving issues such as automatic data transformation when entering data into spreadsheets e.g. gene names converted to dates, dealing with the maximum number of characters in a cell allowed, etc.) and then automatically converted to the deploy version of the database. The parsing of data from these tables to html was done via in-house built software. We also added interactive controls to the html tables such as sorting, pagination, multi-column sorting, search interface and data export to multiple formats by incorporating the Data Tables plugin for jQuery JavaScript library.

The database can be accessed by browsing by type of study, sample (page with clinical data), tissue and type of disease. Furthermore, browsing or searching across all the molecules from the entire database is possible, in which we provide an option of group view or detailed view (provides more molecular information e.g. sequences, mass-to-charge ratios, etc.).

The ability to browse the database through different page menus and jump to others is shown by starting for example in the tissue/fluid source index page (Figure S1) which encloses the disease/condition type, species, number of studies and number of molecules associated with each tissue/fluid source (kidney, urine, blood, etc.) and then jump to the associated omic studies detected in blood (Figure S2), afterwards we can select an experiment dealing with human cohorts in chronic renal insufficiency (CRI) and detected in mononuclear cells. Then, we can navigate to the page for that particular experiment (Figure S3) which contains bibliometric data associated with the research article, a description of the sample characteristics with a link to the clinical data (Figure S5), the main steps in sample preparation, the detection platform used and a list of molecules identified. We can for example select golgin-45 (BLZF1) that was detected increased in expression when compared with healthy subjects in a microarray experiment using peripheral blood mononuclear cells from CRI patients and compare their regulation trend across all the studies, disease conditions, species, and tissue/fluid source from the database (Figure S4).

### Database statistics

The CKDdb database contains 49395 molecule entries (redundant), of which 16885 are unique molecules (non-redundant) from 377 manually curated studies of 230 publications related with CKD (Table 1).

**Figure 3:**
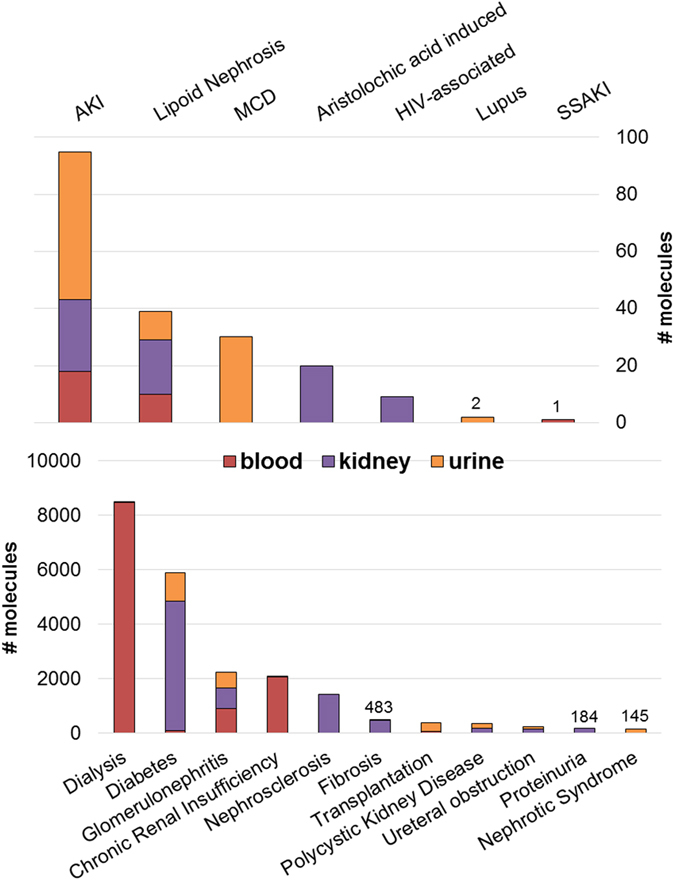
Summary of the number of molecules per disease/condition/treatments and most common tissue/fluid sources (blood, kidney and urine) only for human associated data.

**Figure 4:**
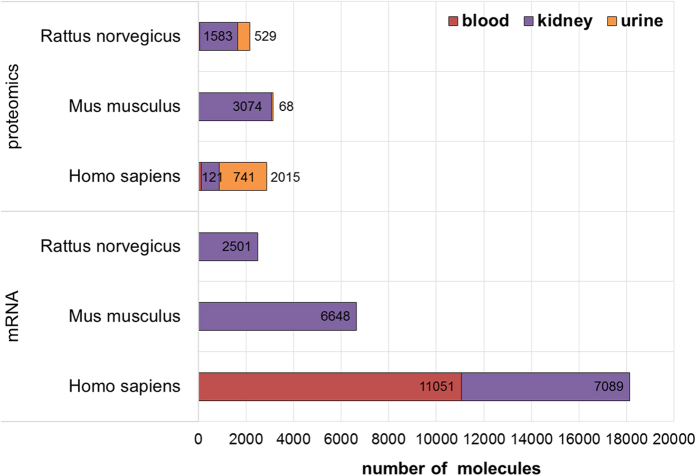
Summary of the number of associated molecules per proteomic and mRNA acquisition methods for the three most represented species within the CKDdb database and per tissue/fluid sources (blood, kidney and urine).

**Figure 5:**
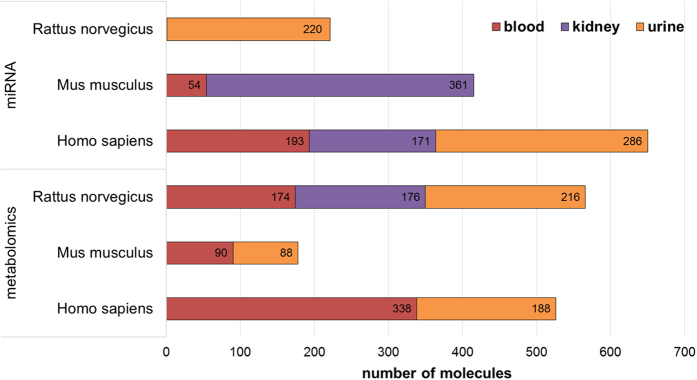
Summary of the number of associated molecules per miRNA and metabolomic acquisition methods for the three most represented species across the CKDdb database and per tissue/fluid sources (blood, kidney and urine).

A summary of the total number of the detected molecules for the most common diseases, conditions and treatments for human studies within CKDdb, which goes across blood, kidney and urine samples, can be found in the Fig. 3. Alongside, the total number of molecules detected per acquisition method and per organism across blood, kidney and urine can be found in Figs 4 and 5. Functionality tag clustering of all the non-redundant data within the CKDdb database was performed (Figure S6) in order to obtain a global view of the content of the database on matter of molecular functionality, which promotes data stratification for an upcoming analysis at the system level.

Hereafter data thresholding (Pvalue: <0.05 and fold-change: down-regulated ≤0.25 and up-regulated ≥4) we used the Cytoscape^14^ plugin ClueGO v2.1.6^15^ to identify the main associated biological processes using gene ontology (GO) of all the non-redundant data within the CKDdb database (Figures S7–S9). We also cross-compared blood, kidney and urine datasets from mRNA and protein studies and in this way identifying the involved pathway(s) terms from KEGG^16^ and WikiPathways^17^, splitting it by regulation trend (Figure S10).

## Utility

The target public for the CKDdb are researchers in the ‘omics field and clinicians that want to perform cross-comparisons among studies and conditions related with CKD. This database can be the starting point for whom that needs to write-up systematic reviews and develop initial hypotheses for experimental testing. To our knowledge this database is the most comprehensive molecular information resource, either in the number of manually curated studies that it contains and as well on the level of detail applied in the description of the experimental setup and linkage with clinical information. This resource is a pristine starting point for investigators who have particular needs of modelling and performing pathway analysis in CKD and associated traits based on molecular expression profiling.

The CKDdb database is able to automatically match orthologous and homologous human genes across a full array of animal models, a feature quite relevant in a systems biology analysis and of grown importance for translation to clinical practice. The ultimate goal of the CKDdb database is to allow the development of pathway/disease models in the framework of CKD.

As a show case of the potential use of the data contained within CKDdb, we first performed an exploratory analysis, based solely on the differential expression of molecules within several kidney diseases and conditions across all the data from the database.

Hereafter, a targeted approach was performed by selecting a specific disease term: chronic renal insufficiency (CRI), which is a synonym for CKD using the medical subject headings (MeSH) terminology as vocabulary control. We selected multiple tissues/fluid sources (blood, urine and kidney) that were characterised in a multitude of ‘omics platforms (Table S1, dataspace description).

### Exploratory analysis: statistical approach based on the expression profiles of the several disease/traits and conditions across the database

#### Data pre-processing

Redundant entries present within the same study were combined by first matching it to our unique identifier system and then using the mean of their expression ratios. Afterwards, we scaled all the data used in the analysis to the [0, 1] interval in order to balance both weight and effects of the fold-change variable from all the disparate number of data sets containing heterogeneous ‘omic platforms and methodologies retrieved from literature.

#### Clustering and Principal Components Analysis (PCA)

**Figure 6:**
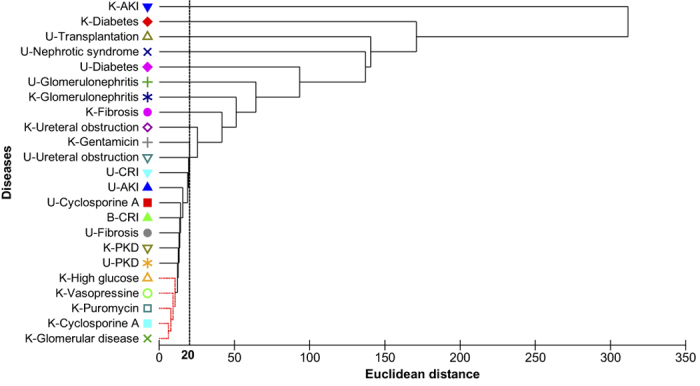
Hierarchical clustering of the molecular expression of microRNA, gene, protein and metabolites by group averaging of the Euclidean distances between renal diseases from multiples tissue and fluid sources. The vertical line represents a level of resemblance of 20%, in which we are able to distinguish two major clusters.

**Figure 7:**
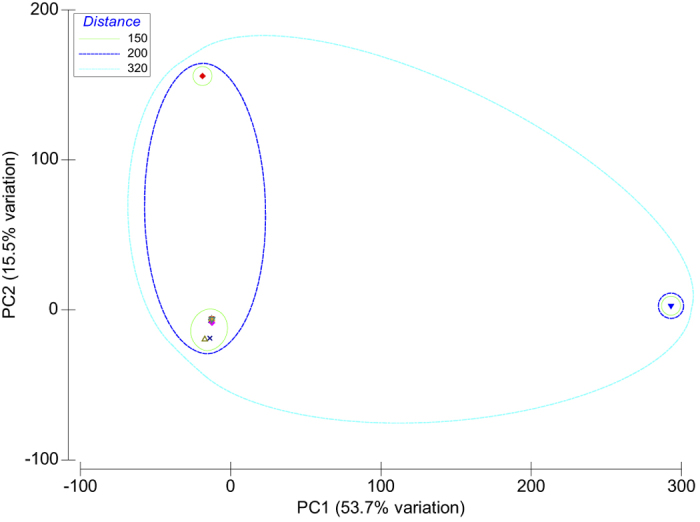
PCA analysis with grouping using hierarchical clustering. K-AKI and K-Diabetes are more correlated with PC1 and PC2 respectively; both components account for more than 69% of the cumulative variation of the data.

As a form of exploratory analysis of the database content we performed hierarchical clustering (Fig. 6) with linkage by group averaging of the Euclidean distances of the most representative traits and conditions present in the CKDdb database based on their expression profiles of the featured molecules (ratios, fold-changes as case against control) in several ‘omic studies. In a similar way, we performed dimensionality reduction by principal component analysis (PCA) of the data having as input the molecular features as rows and traits/conditions as columns (Fig. 7). Then this initial table was transformed into a triangular matrix based on dissimilarity measured as Euclidean distances.

**Figure 8:**
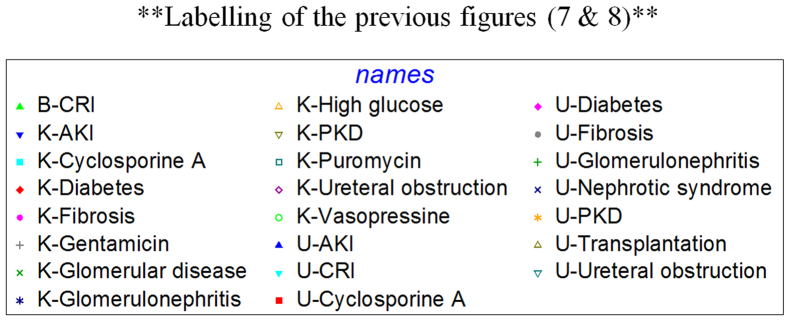
Tissue/fluid source: B-blood, K-kidney, U-urine; Disease/conditions: CRI-chronic renal insufficiency, AKI-acute kidney injury, PKD-polycystic kidney disease.

Based on our exploratory approach we can identify datasets relating to acute kidney injury (AKI) and diabetes both measured in kidney tissue as outliers of the bulk of the data present within CKDdb (Figs 6 and 7, and labels Fig. 8) based on molecular differential expression. Moreover, through dimensionality reduction and based on PC1 and PC2 we can explain 69% of the variation of the CKDdb data (Fig. 7), and as well group datasets based on their molecular expression resemblance.

### Targeted analysis: case study with a subset of the CKDdb database - CRI dataset

#### Data pre-processing

In order to get a focused overview of the associated biological processes and pathways associated with CRI, we used molecular information data detected in three tissues/fluid sources using a multitude of omic platforms captured in 19 human studies (Table S1, for dataspace description). Afterwards we started combining redundant entries (using our unique identifiers system; gene and protein clusters) within the same study by taking the mean of the expression ratios of repeated molecules. Then, we applied a global threshold of 1.3 as fold-change (a 30% increase or decrease of the molecular expression regarding case and control ratios) and a p-value <0.05. Subsequently, a consistency check based on the directionality (down-regulated if <0.77 and up-regulated if >1.3) of the expression molecular profiles across different studies was applied, in order to remove entries with contradictory regulation and merging consistent entries by averaging their ratios. Then, we bipartite the main dataset (5101 molecules/features) into a focused dataset which contains molecules reported more than twice and into an unfocused dataset that handles molecules reported only once in the CRI dataspace. This was done to limit the bias toward specific molecules frequently reported and to ensure than the least reported can have some representativity in our analysis.

#### Functionality tag clustering

The molecular features found within both datasets are globally decreased in their expression levels, with a magnitude around three and two, respectively for the unfocused and focused datasets (Figures S11–S14).

When comparing both datasets based on counting the associated tags that have a certain functionality, we can observe that RIB: ribosome, TCR: T-cell receptor and IGG: Immunoglobulin functionality tags are missing in the focused dataset. The features with the functionality tags MET: metabolite, INH: inhibitor (protease, kinase, other enzymes, pathways) and DEV: development, cell growth, differentiation, morphogenesis within the focused dataset are mainly increased in expression, which follows the opposite overall trend of the dataset. Around 18 and 10% of the total number of features, for the unfocused and focused datasets respectively are features without proper functional annotation, thereby the UK: unknown tag was applied. Functionality tags corresponding at least to 10% of the total number of features are described as ENZ: enzyme, enzymatic properties, UK: unknown and TF: transcription and translation, gene regulation for the unfocused dataset; ENZ: enzyme, enzymatic properties, TF: transcription and translation, gene regulation, TP: transport, storage, endocytosis, exocytosis, vesicles, CS: Cell shape (cytoskeleton, cell adhesion, morphology, cell junction, cellular structures, extracellular matrix) and UK: unknown regarding the focused dataset (Figures S11–S14). This primary approach is helpful in the way that it provides basic information characterising the functional role of the molecules within our datasets, since each molecule just has a unique tag. Therefore, it helps the data analyst to reinforce his strength towards particular areas e.g. regulatory networks (in case of transcriptional factors, miRNAs and their target genes), signalling pathways, metabolic pathways, etc.).

#### Gene ontology and pathway term clustering

The use of multiple gene ontologies (GO) and pathway terms allowed us to identify based on the expression of our input genes/proteins from the focused dataset, significant down-regulated clusters modulating signalling (Thyroid-stimulating hormone: TSH and Brain-derived neurotrophic factor: BDNF) pathways, regulation of microtubule depolymerisation, aminoglycan metabolic processes and protein N-linked glycosylation (Figure S15). On the other hand, significant up-regulated clusters of processes related with binding and uptake of ligands by scavenger receptors and as well linked with complement and coagulation cascades (Figure S15).

#### Molecular clustering based on protein-protein interactions (PPI’s)

Unfortunately, not every protein/gene has annotated functions; therefore in order to cover the not so well annotated molecules, we performed protein-protein interactions with both datasets (focused plus unfocused) with the representation of representing their respective molecular expression in GeneMania^18^ (Figure S16).

Afterwards, we used as input molecules the ones from the initial pathway term clustering of the TSH and BDNF signalling pathways (Figure S15) and performed as well PPI’s analysis (Figure S17) in GeneMania^18^, in order to potentially reveal newly connected molecules from the enrichment.

#### Regulatory networks: miRNA-target genes

We performed the association of down-regulated microRNAs and their up-regulated gene targets (Figure S18A) using miRNAs from both datasets and gene/proteins from the focused dataset via Cytoscape^14^ software and ClueGO+CluePedia application^14^,^15^,^19^. Additionally, we did the linkage of miRNAs to target genes (Figure S18A) and their associated processes and pathways terms (Figures S18B and S18C). We found that the hsa-miR-200a-3p modulates both C4B (Complement C4-B) and TTR (Transthyretin) that are associated respectively, with complement and coagulation cascades and the metabolism of fat-soluble vitamins (Figures S18B and S18C). Interestingly, the other members of the miR-200 family (200b and 200c), and as well the hsa-miR-378a-5p were found to modulate TTR on the same way. The hsa-miR-708-5p modulates IDS (Iduronate 2-sulfatase), FABP3 (Fatty acid-binding protein, heart) and AMBP (Protein AMBP), with the latter being associated with binding and uptake of ligands by scavenger receptors.

#### Interactome matching: gene-enzyme-reaction-metabolite

Using our focused dataset having as input genes/proteins and metabolites, we found an association with the Proteoglycan biosynthesis (Figure S19A), *N*-glycan biosynthesis (Figure S19B) and *O*-glycan biosynthesis (Figure S19C) via Cytoscape^14^ software and Metscape application^14^,^20^.

#### Mapping of the molecular features into existing pathway maps

We mapped our molecules with their respective regulation ratios onto existing pathway maps using WikiPathways^17^ via PathVisio software^21^ and as well onto the AKI full pathway map^22^ and then merged both and removed the features not mapped. In first instance we mapped firstly our “focused” dataset to give emphasis to the most relevant pathways and then added the “unfocused” dataset with the purpose of gap-filling (Figure S20).

We found a general down-regulation of regulatory proteins involved in the cytoskeletal organisation, particularly in the process of microtubule assembly and stability (e.g. APC, APC2, DPYSL2, MAPT, KIF4A, NEFM, and VIM). Moreover, regulatory proteins related with the assembly and stability of actin at the filopodia and lamellipodia level was found down-regulated.

Know hallmarks for chronic kidney disease establishment and progression as extracellular matrix remodelling via degradation of collagenases by metalloproteinases can be observed in our merged pathway map and may serve as a positive control of our analysis.

The SMADs related intracellular proteins (in our case SMAD2 and SMAD7) were found both down-regulated, which can potentially lead to disruption of downstream gene transcription.

Additionally, decreased expression of BDNF and TSH receptor was found within our dataset, which at the first view seemed to be a mere artefact.

On the other hand, the metabolic cascades within the mapped molecules onto our pathway map had too many gaps and therefore our choice was to not include it in our analysis.

#### Merging results and overall interpretation

Taking all together the results from gene ontology and pathway term clustering, plus the interactome based on PPIs, matching miRNAs to target genes and linking genes to metabolites through the analysis of metabolic pathways, it leads to the description as a whole of the several individual datasets collected for CRI. Thereby, clustering based on GO and pathway terms, combined with the mapped molecules onto pathways lead to show how the regulation of microtubules and particularly actin assembly and stabilization were modulated taking into account the fold-change of the involved molecules. Microtubules have active roles in several biological processes, including cell division and intracellular transport. They are one of the principal cytoskeletal components of major podocyte processes, playing a key role in podocyte morphogenesis, podocyte process outgrowth, branching, and elongation. Here, we observed an inhibition of the regulation of microtubule disassembly, concomitantly with actin disassembly and stabilization at the filopodia and lamellipodia level, with the latter leads to the decrease of cell motility and migration and therefore promoting focal adhesion formation^23^,^24^.

A recent study made mention that BDNF has the potential to repair podocyte damage by an increase of actin assembly mediated in a regulatory way by miRNAs^25^. Concomitantly, it was reported that patients undergoing hemodialysis have lower levels of circulating BDNF^26^. Additionally, functional thyroid disorders seems to be more prevalent within patients with chronic renal failure undergoing hemodialysis when compared with healthy population^27^,^28^.

## Discussion

Repositories of general scope for transcriptomics such as GEO^7^,^29^ and EBI Expression Atlas^8^ are a source of both raw and processed data, and as well metadata submitted by the research community. In the GEO case, it also provides tools for analysing and visualising data, the GEO2R, an R-based web-application.

Other initiatives as the Multi-Omics Profiling Expression Database (MOPED) stores and displays molecular expression data from transcriptomics and proteomics studies across organisms, tissues and conditions. The provenience of the mRNA expression data is originally sourced from GEO raw files that are then reanalysed using their own analysis workflows and filtering criteria. On the other hand, proteomic raw data is usually acquired by free submission of the research community and then also being subjected to review and reanalysed by the MOPED team^30^.

The Urinary Protein Biomarker Database is focused in proteins found in urine that can potentially serve as biomarkers in a disease. The authors went through the existing literature in that particular subject and collected information of both proteins and diseases in humans and model organisms. To ensure the highest quality manual review of all extracted information was then performed^31^.

Specific databases regarding molecular expression profiling in renal diseases are still scarce, nevertheless initiatives like Nephroseq, a web-based platform that integrates gene expression data sets with clinical data for kidney diseases more focused on data visualization is available at http://www.nephroseq.org. A similar database derived from data mining of GEO database is the Renal Gene Expression Database (RGED), which allows querying expression profiling of genes in the topic of kidney diseases^32^. The majority of these databases are only focused in one or maximum two ‘omic platforms, lacking a multi-omics database that unifies the bulk of the scattered single ‘omics repositories. The Kidney and Urinary Pathway Knowledge Base (KUPKB) gave the first step to fill this gap, mining and collecting information of about 220 data sets across miRNA, mRNA, protein and metabolites in urine and kidney tissues^33^. Nevertheless, KUPKB only provides the molecular expression as nominal tags like ‘medium’, ‘strong’, ‘up’, ‘down’, ‘absent’, and ‘present’ which raises additionally difficulties in a forward systems level analysis that requires numerical data. Likewise, relevant data descriptor fields are missing such as the description of the experimental setup, statistical scores, fold-change cut-offs, type of omic platform and association with clinical data.

Systems biology requires comprehensive data at all molecular levels, hence using a data-driven approach based in molecular expression profiling of a disease and/or disease states could be a promising approach to find potential body fluid-accessible biomarkers and therapeutic targets. Nevertheless, mining the increasing data across different labs in an efficient way is still a big challenge which affects the efficiency of selecting useful molecular candidates and it results in the accumulation of redundant and false identified molecules. And those will ultimately populate the increasing medical literature and will become a source for biased information and thereby a burden for decision making with respect to candidate biomarkers.

The CKDdb (www.padb.org/ckdbd) was developed as a database to store; query and ultimately to pave the way in the development of disease models from publically available multi-omics data sets in order to bypass the challenges associated with handling and integration of heterogeneous big data. The database provides the researchers to have access to all the high-throughput data available from studies in both CKD and ‘omics platforms together with detailed information regarding molecular expression, functional annotation and as well linkage to clinical data.

CKDdb can be used extensively by researchers in the ‘omics field and by clinicians to cross-compare among studies and conditions, which facilitates the writing-up of systematic reviews and plays an important role in hypotheses generation. To our knowledge this database is the most comprehensive molecular information resource in characterising CKD-related experiments and model systems.

This database is primarily aimed to allow disease pathway analysis through a systems approach in order to yield biological meaning by integrating all existing information and therefore has the potential to unravel and gain an in-depth understanding of the key events that modulate CKD onset and progression.

## Methods

### Database construction

#### Data mining

The content of the database is based on manual extraction of data from available literature on the topic of CKD and ‘omics technologies. We used several strings in PubMed in order to cover the topic as much as possible (e.g. “Renal Insufficiency, Chronic” [Mesh] AND (miRNA OR genomic* OR proteomic* OR metabolomic*) NOT review). The initial effort in data gathering started to be specifically about CKD, however with the development of the database we realised that being dependent only on a unique trait such as CKD and having as a final goal the development of a disease/pathway model wouldn’t be adequate due to the multifactorial nature of CKD in which other disorders could play a crucial role in triggering this disease. Thus, we modified our search string (e.g. “Kidney Diseases” [Mesh] AND (miRNA OR genomic* OR proteomic* OR metabolomic*) NOT (review OR neoplasms) in order to be broader, capturing this way other related traits that could contribute for CKD onset and progression (e.g. focal segmental glomerulosclerosis, diabetic nephropathy, fibrosis, nephrotic syndrome, etc.).

#### Data curation

The step of data curation plays a central role in the mainstream of a database development, in which the quality of the collected data is verified by comparing it with similar studies and by assessing the relevance of the parameters used in the detection of a molecule or molecule population (e.g. statistical thresholds, normalisation method, applied cut-offs, validation steps, reference databases used for matching, etc.).

As initially expected the number of redundant molecules seems to be directly proportional to the increase in the number of studies and then it reaches a plateau (which is equivalent to reach the point of the current knowledge about the transcriptome, proteome and metabolome of a specific species).

Hereafter, data was converted to our internal identifiers: clustered sequences and orthologs (CluSO) and ortholog mapping resource (OMAP) from the Pan-omics Analysis Database (PADB) initiative. The CKDdb is fully integrated into other databases held within the PADB infrastructure. These internal identifiers allow us to deal with the high heterogeneity of the data sourced from multi-omic studies of available literature.

We only kept statistically significant and differently expressed molecules e.g. p-value <0.05, and fold-changes (FC), in which the selected threshold is dependent on the detection method used by the authors i.e. transcriptomics FC ≥ 2; proteomics and metabolomics FC ≥ 1.3.

## Additional Information

**How to cite this article**: Fernandes, M. and Husi, H. Establishment of a integrative multi-omics expression database CKDdb in the context of chronic kidney disease (CKD). *Sci. Rep.* **7**, 40367; doi: 10.1038/srep40367 (2017).

**Publisher's note:** Springer Nature remains neutral with regard to jurisdictional claims in published maps and institutional affiliations.

## Supplementary Material

Supplementary Information

## Figures and Tables

**Table 1:** Current stats of the CKDdb database.

No. of total entries with extended information: 12462	No. of unique (non-redundant) molecules: 16885	Total no. of samples in Demographics file: 187
No. of unique studies with extended information: 12	Total no. of studies in Study reference file/Table: 377	No. of unique samples in Demographics file: 187
Total no. of molecule entries (redundant): 49395	Total no. of unique studies: 377	No. of entries in Peptidomics file: 1396
