# Supplementary material for: Establishment of a integrative multi-omics expression database CKDdb in the context of chronic kidney disease (CKD)
Source: Sci Rep. 2017 Jan 12;7:40367. doi: 10.1038/srep40367 (PMC5227717; doi:10.1038/srep40367)

Supplementary material

Establishment of a integrative multi-omics expression database CKDdb in the context of chronic kidney disease (CKD)

Marco Fernandes [a] and Holger Husi [a]

[a] Institute of Cardiovascular and Medical Sciences, University of Glasgow, BHF Glasgow Cardiovascular Research Centre, 126 University Place, Glasgow, G12 8TA, UK

m.fernandes.1@research.gla.ac.uk; Holger.Husi@glasgow.ac.uk

Corresponding author:

Dr Holger Husi, Institute of Cardiovascular and Medical Sciences, University of Glasgow, Joseph Black Building, Room B2-21, Glasgow, G12 8QQ, UK

Tel.: 0044 141 330 6210

FAX: 0044 141 330 7394

Email: Holger.Husi@glasgow.ac.uk

Content

Construction steps........5

Page-view illustrations of the CKDdb database........6

Functionality tag clustering – [CKDdb]........11

Term clustering – global – CKDdb database........12

Term clustering – pathways (KEGG and WikiPathways)........17

Targeted analysis: case study with a subset of the CKDdb database - CRI datasets........18

Functionality tag clustering – [focused dataset]........19

Functionality tag clustering – [unfocused dataset]........20

Gene ontology (GO) and pathway term clustering [Focus dataset]........21

Molecular clustering based on protein-protein interactions (PPI's): GeneMania | [both datasets]........22

Molecular clustering based on protein-protein interactions (PPI's): GeneMania | [Focus dataset]........23

Regulatory networks: microRNAs-target genes........24

Interactome matching: gene -metabolite| [Focus dataset]........26

Mapping of the molecular features into existing pathway maps........27-28

Figure index

Figure S1 Index page of the tissue/source list of the CKDdb database. The fields of disease, species, number of studies and number of molecules per each tissue/fluid (kidney, urine, blood, etc.) source are described. 6
Figure S2 Index page for the omic studies detected in blood tissue/source. The fields of species, component, number of molecules, disease, and PMID/DOI are described per each study.. 7
Figure S3 Structure of the CKDdb at the study page view. First headers enclose the main bibliometric data associated with a research article. The second and third headers are with respect to the description of the sample itself and several steps in their processing till reach molecule identification. The fourth header corresponds to the incoming molecules from the previous step. It lists all the molecules identified in the study with the associated statistical scores, fold-change and regulation.. 8
Figure S4 View of the of a specific molecule entry in CKDdb. In the first header is displayed the linkage to external databases. Following it we have represented the associated function and classification and as well our own type of tag for functional classification (derived from PADB). The third header corresponds to the number of occurrences of the molecule across all the studies, disease conditions, species, and tissue/fluid source. 9
Figure S5 - Clinical data (demographics page view) is also represented in the CKDdb database and is linked with the study page and vice-versa.. 10
Figure S6 - Functionality tag clustering of the total differentially expressed molecules (fold-change: down-regulated ≤0.66 and up-regulated ≥1.5 and Pvalue <0.05) for the CKDdb database. ENZ: enzyme, enzymatic properties; UK: unknown; CS: cell shape (cytoskeleton, cell adhesion, morphology, cell junction, cellular structures, extracellular matrix); TF: transcription and translation, gene regulation; TP: transport, storage, endocytosis, exocytosis, vesicles; TM: transmembrane; SIG: signalling; RCP: receptor; MIR: microRNA; MOD: modulator, regulator; CNL: channel; INH: inhibitor (protease, kinase, other enzymes, pathways); CC: cell cycle (turnover, mitosis, meiosis); MET: metabolite; RIB: ribosome; DEV: development, cell growth, differentiation, morphogenesis; CHA: chaperone, chaperonin; DIS: disease; IGG: immunoglobulin. 11
Figure S7 - GO group functionality (biological process) and molecular regulation for blood (only human data across all diseases present in the database). GO term/pathway network connectivity (kappa score): 0.4; GO term fusion; pathways/term pV≤0.01. Data thresholding, Pvalue: <0.05 and Fold-change: down-regulated ≤0.25 (green nodes) and up-regulated ≥4 (red nodes). The pie-chart represents cluster#1 – down-regulated with their associated GO terms... 12
Figure S8 - GO group functionality (biological process) and molecular regulation for the kidney tissue (only human data across all diseases present in the database). GO term/pathway network connectivity pathway network connectivity (kappa score): 0.4; GO term fusion; pathways/term pV≤0.01. Data thresholding, Pvalue: <0.05 and Fold-change: down-regulated ≤0.25 (green nodes) and up-regulated ≥4 (red nodes). The pie-chart represents cluster#2 – up-regulated with their associated GO terms.. 14
Figure S9 - GO group functionality (biological process) and molecular regulation for urine (only human data across all diseases present in the database). GO term/pathway network connectivity pathway network connectivity (kappa score): 0.4; GO term fusion; pathways/term pV≤0.01. Data thresholding, Pvalue: <0.05 and Fold-change: down-regulated ≤0.25 (green nodes) and up-regulated ≥4 (red nodes). The pie-chart represents respectively cluster#1 – down-regulated and cluster#2 – up-regulated with their associated GO terms. 16
Figure S10 ClueGO analysis of the associated pathway(s) terms from KEGG and WikiPathways. Cross-comparison between kidney, blood and urine datasets. 17
Figure S11 - Functionality tag clustering of the total differentially expressed molecules (N=241, fold-change <0.77 & >1.3 and Pvalue <0.05) for the focused dataset. ENZ: enzyme, enzymatic properties; TF: transcription and translation, gene regulation; TP: transport, storage, endocytosis, exocytosis, vesicles; CS: Cell shape (cytoskeleton, cell adhesion, morphology, cell junction, cellular structures, extracellular matrix); UK: unknown; MET: metabolite; RCP: receptor; SIG: signalling; INH: inhibitor (protease, kinase, other enzymes, pathways); TM: transmembrane; MOD: modulator, regulator; CC: cell cycle (turnover, mitosis, meiosis); DIS: disease; CNL: channel; SCA: scaffolder, docking, adaptor; DEV: development, cell growth, differentiation, morphogenesis; MHC: major histocompatibility complex component/protein cluster (MHC, HLA); MIR: microRNA; CHA: chaperone, chaperonin. 19
Figure S12 - Functionality tag clustering of the down (N=169) and up-regulated (N=72) molecules (fold-change ≤0.77 & >1.3 and Pvalue <0.05) for the focused dataset. 19
Figure S13 - Functionality tag clustering of the total differentially expressed molecules (N=4860, fold-change <0.77 & >1.3 and Pvalue <0.05) for the unfocused dataset. ENZ: enzyme, enzymatic properties; UK: unknown; TF: transcription and translation, gene regulation; CS: Cell shape (cytoskeleton, cell adhesion, morphology, cell junction, cellular structures, extracellular matrix); TM: transmembrane; TP: transport, storage, endocytosis, exocytosis, vesicles; RCP: receptor; MET: metabolite; SIG: signalling; CC: cell cycle (turnover, mitosis, meiosis); CNL: channel;

MOD: modulator, regulator; DEV: development, cell growth, differentiation, morphogenesis; RIB: ribosome; DIS: disease; INH: inhibitor (protease, kinase, other enzymes, pathways); CHA: chaperone, chaperonin; SCA: scaffolder, docking, adaptor; MIR: microRNA; TCR: T-cell receptor; IGG: Immunoglobulin; MHC: major histocompatibility complex component/protein cluster (MHC, HLA). 20
Figure S14 - Functionality tag clustering of the down (N=3672) and up-regulated (N=1188) molecules (fold-change <0.77 & >1.3 and Pvalue <0.05) for the unfocused dataset. 20
Figure S15 Overall view of the term clustering based on molecular regulation (down: green, up: red) from GO (Biological process, immune system and molecular function) and pathway term clustering (KEGG, Reactome and WikiPathways) of the focused dataset within CRI and human data, across multi-tissues and fluid sources (blood, urine and kidney). Network merging (union) of GO terms (Biological Process, Molecular Function and Cellular component), and Pathway terms (Reactome, KEGG, and WikiPathways) into a global network performed in ClueGO. 21
Figure S16 Molecular clustering based on protein-protein interactions (PPI's) and regulation using both datasets (focused + unfocused) plus enrichment (grey nodes) in GeneMania. 22
Figure S17 Molecular clustering based on protein-protein interactions (PPI's) and regulation using both datasets (focused + unfocused) plus enrichment (grey nodes) in GeneMania. .... **Error! Bookmark not defined.**
Figure S18 Association of down-regulated microRNAs and their up-regulated gene targets (A) using miRNAs from both datasets and gene/proteins from the focused dataset via Cytoscape software and ClueGO+CluePedia application. Linkage of miRNAs to targets and their associated pathways terms B and C. 25
Figure S19 - Association of genes/proteins and metabolites for the Proteoglycan biosynthesis (A), N-glycan biosynthesis (B) and O-glycan biosynthesis (C) using our focused dataset via Cytoscape software and Metscape application. 26
Figure S20 Mapping of the molecular features into existing pathway maps........ 27

Table index

Table S1 Dataspace description of the 19 studies used in our analysis. EXPREF: is the PMID identifier preceded by "Exp". NA: no mention within the study at least on a straightforward manner. Type: states the molecules associated in the study; G: gene, M: metabolite; MIR: miRNA; P: protein. Only human studies were used in this analysis. 18

Construction steps

Data mining

The content of the database is based on manual extraction of data from the available literature on the topic of CKD and ‘omics technologies. We used several strings in PubMed in order to cover the topic as much as possible (e.g. "Renal Insufficiency, Chronic"[Mesh] AND (miRNA OR genomic* OR proteomic* OR metabolomic*) NOT review). The initial effort in data gathering started to be specifically about CKD, however with the evolving of the database we realised that being dependent only on a unique trait such as CKD and having as a final goal the development of a disease/ pathway model wouldn’t be adequate due to the multifactorial nature of CKD in which other disorders could play a crucial role in triggering this disease. Thus, we modified our search string (e.g. "Kidney Diseases"[Mesh] AND (miRNA OR genomic* OR proteomic* OR metabolomic*) NOT (review OR neoplasms) in order to be broader, capturing this way other related traits that could contribute for CKD onset and progression (e.g. FSGS, DN, fibrosis, nephrotic syndrome, etc.).

Data curation

The step of data curation plays a central role in the mainstream of a database development, in which the quality of the collected data is verified by comparing it with similar studies and by assessing the relevance of the parameters used in the detection of a molecule or molecule population (e.g. statistical thresholds, normalisation method, applied cut-offs, validation steps, reference databases used for matching, etc.). Hereafter, data is converted to our internal identifiers: CluSO and OMAP from the Pan-omics Analysis Database (PADB) initiative. The CKDdb is fully integrated into other databases held within the PADB framework. These internal identifiers allow us to deal with the high heterogeneity of the data sourced from multi-omic studies of the available literature.

We only kept statistically significant and differently expressed molecules e.g. p-value <0.05, and fold-changes (FC), in which the selected threshold is dependent on the detection method used by the authors i.e. transcriptomics FC$\geq$ 2; proteomics and metabolomics FC$\geq$ 1.3.

Database structure and deployment

The CKDdb database is based on a NoSQL-type data structure, whereby source data is manipulated in spreadsheets followed by parsing to convert the data into pre-assembled and interlinked html files. There are 5 different tables relating to demographics, experimental setup, statistically found significant molecules, extended molecular information, and peptidomics data (peak profiling data). All of these tables follow the standard database nomenclature and structure, i.e. they are all linked to each other and to external databases such as UniProt/ SwissProt, ChEBI, NCBI, miRbase, EMBL and Unigene. Thus, in this way the customised parser allows to emulate a database system by pre-assembling query outputs as e.g. grouping based on disease/ tissue/ molecule/ etc.

For a matter of simplicity and usability, all collected data was stored in spreadsheets (but avoiding and solving issues such as automatic data transformation when entering data into spreadsheets e.g. gene names converted to dates, dealing with maximum number of characters in a cell, etc.) and then automatically converted to the deploy version of the database. The conversion of data from these tables to html was done via an in-house built software. We also added interactive controls to the html tables such as sorting, pagination, multi-column sorting and search interface by incorporating the DataTables plugin for jQuery JavaScript library.

Page-view illustrations of the CKDdb database

CKDdb - Tissue list

Copy | Print | Export table | Show / hide columns

Show 10 entries

Search:

Tissue / Source	Disease	Species	# of studies	# of molecules
kidney	Diabetes (diabetic nephropathy), Polycystic_Kidney_Disease (autosomal recessive polycystic kidney disease), Glomerulonephritis (Thy_1_nephritis), Transplantation (Allograft Rejection allo. CBA D5), Transplantation (Allograft Rejection iso.CBA D5), proteinuria (proteinuric nephropathies), IAKI (Ischemic acute kidney injury), remnant_kidney_model (remnant kidney model), Diabetes (diabetic nephropathy in Type 1 diabetes), nephrin_knockout (nephrin_knockout), Glomerulonephritis (lupus nephritis), ureteral_obstruction (unilateral ureteral obstruction), kidney_calculi (Calcium oxalate monohydrate in vitro model), Polycystic_Kidney_Disease (Autosomal Dominant Polycystic kidney disease), Urination_Disorders (proteinuria), proteinuria (proteinuria), Polycystic_Kidney_Disease (Autosomal dominant polycystic kidney disease), Cyclosporine_A_induced (Ciclosporine A in vitro model), Fibrosis (Interstitial fibrosis and tubular atrophy), High_glucose_induced (High glucose in vitro model), Fibrosis (TGFbeta in vitro model), Diabetes (Diabetic Nephropathy), Glomerulonephritis (IgA nephropathy), Glomerulonephritis (Glomerulonephritis), Nephrosclerosis (glomerulosclerosis), IRI (ischemic injury model), Polycystic_Kidney_Disease (Autosomal Dominant Polycystic Kidney disease), Urination_Disorders (transgenetic proteinuria), Polycystic_Kidney_Disease (polycystic kidney disease), Glomerulonephritis (FSGS + COLL), Vasopressine_induced (Vasopressine in vitro model), Diabetes (type 2 diabetes), Glomerulonephritis (glomerular hyperfiltration), glomerular_disease (glomerular disease), Diabetes (diabetic nephropathy in T1D), Diabetes (diabetic kidney disease), Glomerulonephritis (membranous nephropathy), Lipoid_Nephrosis (minimal change disease), Glomerulonephritis (focal segmental glomerulosclerosis), Gentamicin_induced (Gentamicin-induced nephropathy), ureteral_obstruction (unilateral hydronephrosis), Glomerulonephritis (mesangial proliferative glomerulonephritis), Diabetes (type 2 diabetic nephropathy), AKI (AKI), Fibrosis (Fibrosis), Glomerulonephritis (Passive Heymann nephritis), Chronic_Renal_Insufficiency (mouse_CKD_model), Diabetes (streptozotocin-induced diabetic), Dialysis (Peritonitis), HIV_associated (HIV-associated nephropathy in vitro), Glomerulonephritis (mesangioproliferative glomerulonephritis), Chronic_Renal_Insufficiency (chronic renal failure), cisplatin_induced (cisplatin-induced nephrotoxicity), Fibrosis (renal interstitial fibrosis), Glomerulonephritis (FSGS), ureteral_obstruction (renal tubulointerstitial injury), Glomerulonephritis (chronic glomerulonephritis), IRI (Ischemia-Reperfusion Injury), Chronic_Renal_Insufficiency (early_stage_CKD), nephropathies (collagen IV nephropathies), ureteral_obstruction (kidney obstruction in vitro model), IRI (ischemia-reperfusion injury), Puromycin_induced (Puromycin nephropathy model), Aristolochic_acid_induced (Aristolochic Acid Nephropathy), Fibrosis (kidney fibrosis), IRI (Bilateral Renal Ischemia Reperfusion), ureteral_obstruction (obstructive uropathy), Diabetes (diabetic nephropathy induced by Streptozotocin), Diabetes (diabetic nephropathy treated with Phlorizin), Glomerulonephritis (IgA nephropathy (with endocapillary proliferation (E1))), IRI (ischemia/reperfusion injury), Chronic_rejection (Chronic_rejection), Chronic_Renal_Insufficiency (CKD), Glomerulonephritis (membranous_nephropathy)	Homo sapiens, Mus musculus, Rattus norvegicus, Canis lupus familiaris	172	31724
urine	Transplantation (acute renal allograft injury), Glomerulonephritis (IgA nephropathy), Glomerulonephritis (membranoproliferative glomerulonephritis model), AKI (AKI), Glomerulonephritis (membranous nephropathy), Chronic_Renal_Insufficiency (Chronic kidney disease), Diabetes (Diabetic Nephropathy), Glomerulonephritis (Passive Heymann Nephritis), Glomerulonephritis (focal segmental glomerulosclerosis), Glomerulonephritis (adriamycin nephropathy and Thy1.1 induced glomerulonephritis), Diabetes (diabetic nephropathy), Fibrosis (Interstitial fibrosis and tubular atrophy), Transplantation (acute renal allograft rejection), ureteral_obstruction (unilateral uteropelvic junction obstruction), Cyclosporine_A_induced (cyclosporine A nephrotoxicity), ureteral_obstruction (unilateral ureteral obstruction), Glomerulonephritis (basement membrane nephropathy), Chronic_Renal_Insufficiency (CKD_stage_4), lupus (systemic lupus erythematosus), Glomerulonephritis (glomerular kidney disease), Glomerulonephritis (lupus nephritis), Diabetes (type 2 diabetic nephropathy), ureteral_obstruction (congenital bilateral hydronephrosis), HUPM), Polycystic_Kidney_Disease (Autosomal Dominant Polycystic Kidney disease), Gentamicin_induced (chronic sensitization to gentamicin), Diabetes (diabetes mellitus), Glomerulonephritis (membranous glomerulonephritis), Glomerulonephritis (focal glomerulosclerosis), Nephrotic_Syndrome (Idiopathic Nephrotic Syndrome), Diclofenac_induced (diclofenac-induced renal injury), Diabetes (type 2 diabetes), Transplantation (chronic allograft dysfunction), Diabetes (type 1 diabetes without retinopathy or nephropathy), Diabetes (type 1 diabetes with retinopathy), Diabetes (type 1 diabetes with retinopathy and nephropathy), Diabetes (type 1 diabetes with retinopathy vs type 1 diabetes without retinopathy or nephropathy), Diabetes (type 1 diabetes with retinopathy and nephropathy vs type 1 diabetes without retinopathy or nephropathy), Diabetes (type 1 diabetes with retinopathy and nephropathy vs type 1 diabetes with retinopathy), Glomerulonephritis (FSGS/MCD), Glomerulonephritis (crescentic glomerulonephritis (CrGN)), Diabetes (type 1 diabetes (w/ microalbuminuria)), Glomerulonephritis (Primary focal segmental glomerulosclerosis), MCD (minimal change disease), healthy (healthy), Diabetes (Type 2 diabetes mellitus), cisplatin_induced (cisplatin-induced nephrotoxicity), cisplatin_induced (cisplatin-induced kidney injury), Nephrotic_Syndrome (paediatric idiopathic nephrotic syndrome), Diabetes (type 2 diabetes without nephropathy), Diabetes (type 2 diabetes with retinopathy), Diabetes (diabetes with overt diabetic nephropathy), Lipoid_Nephrosis (minimal change disease (MCD))	Homo sapiens, Mus musculus, Rattus norvegicus, Macaca mulatta	123	3748
blood	Transplantation (Acute Renal Allograft Rejection), Chronic_Renal_Insufficiency (Chronic kidney disease), Dialysis (hemodialysis), Diabetes (Gestational Diabetes), Glomerulonephritis (IgA nephropathy), Chronic_Renal_Insufficiency (chronic kidney failure), Chronic_Renal_Insufficiency (CKD_stage_4), Fibrosis (renal interstitial fibrosis), SSAKI (severe septic shock-associated kidney injury), Chronic_Renal_Insufficiency (adenine-induced chronic renal failure), AKI (AKI), Doxorubicin_induced (Doxorubicin-induced nephropathy), Glomerulonephritis (membranous nephropathy, HUPM), Glomerulonephritis (FSGS), Chronic_Renal_Insufficiency (CKD_stage_3), Nephrotic_Syndrome (Idiopathic Nephrotic Syndrome), Diabetes (diabetic nephropathy), Transplantation (Chronic Antibody-Mediated Rejection), Chronic_Renal_Insufficiency (End-stage renal failure), healthy (healthy), Dialysis (Peritoneal dialysis), Glomerulonephritis (membranous nephropathy), Diabetes (diabetes mellitus), Chronic_Renal_Insufficiency (end-stage renal disease), Diabetes (streptozotocin-induced diabetes), Diabetes (type 2 diabetes), Diabetes (Type 2 diabetes mellitus), IRI (Bilateral Renal Ischemia Reperfusion), IRI (ischemia/reperfusion injury), Glomerulonephritis (focal segmental glomerulosclerosis), Lipoid_Nephrosis (minimal change disease (MCD)), Chronic_Renal_Insufficiency (CKD)	Rattus norvegicus, Homo sapiens, Mus musculus, Macaca mulatta	69	12179
macrophage	Glomerulonephritis (lupus nephritis), inflammation (chronic_inflammation)	Mus musculus	3	647
aortas	Diabetes (Diabetes)	Mus musculus	2	372
corpora	Diabetes (Diabetes Mellitus-associated Erectile Dysfunction)	Rattus norvegicus	2	114

Figure S1 Index page of the tissue/source list of the CKDdb database. The fields of disease, species, number of studies and number of molecules per each tissue/fluid (kidney, urine, blood, etc.) source are described.

CKDdb - Tissue ID blood

All molecules in this tissue

Copy | Print | Export table | Show / hide columns

Show 100 entries

Search:

Study ID	Species	Component	# of molecules	Disease	PMID/DOI
Exp16083269	Rattus norvegicus	serum	18	Transplantation (Acute Renal Allograft Rejection)	16083269
Exp19698090a	Homo sapiens	mononuclear cells	725	Chronic_Renal_Insufficiency (Chronic kidney disease)	19698090
Exp19698090b	Homo sapiens	mononuclear cells	8265	Dialysis (hemodialysis)	19698090
Exp20348205a	Rattus norvegicus	plasma	32	Diabetes (Gestational Diabetes)	20348205
Exp20348205b	Rattus norvegicus	plasma	2	Diabetes (Gestational Diabetes)	20348205
Exp20485333	Homo sapiens	whole	183	Glomerulonephritis (IgA nephropathy)	20485333
Exp20613759	Homo sapiens	plasma	60	Chronic_Renal_Insufficiency (chronic kidney failure)	20613759
Exp20812764	Homo sapiens	plasma	67	Transplantation (Acute Renal Allograft Rejection)	20812764
Exp21183621	Homo sapiens	serum	38	Chronic_Renal_Insufficiency (Chronic kidney disease)	21183621
Exp21569504a	Homo sapiens	plasma	5	Chronic_Renal_Insufficiency (Chronic kidney disease)	21569504
Exp21569504b	Homo sapiens	plasma	9	Chronic_Renal_Insufficiency (Chronic kidney disease)	21569504
Exp21569504c	Homo sapiens	plasma	3	Chronic_Renal_Insufficiency (Chronic kidney disease)	21569504
Exp21891774a	Homo sapiens	plasma	6	Chronic_Renal_Insufficiency (CKD_stage_4)	21891774
Exp22027911a	Rattus norvegicus	serum	10	Fibrosis (renal interstitial fibrosis)	22027911
Exp22027911b	Rattus norvegicus	serum	19	Fibrosis (renal interstitial fibrosis)	22027911
Exp22027911c	Rattus norvegicus	serum	20	Fibrosis (renal interstitial fibrosis)	22027911
Exp22098946	Homo sapiens	whole	1	SSAKI (severe septic shock-associated kidney injury)	22098946
Exp22133066	Rattus norvegicus	serum	11	Chronic_Renal_Insufficiency (adenine-induced chronic renal failure)	22133066
Exp22429878	Homo sapiens	serum	18	AKI (AKI)	22429878

Figure S2 Index page for the omic studies detected in blood tissue/source. The fields of species, component, number of molecules, disease, and PMID/DOI are described per each study.

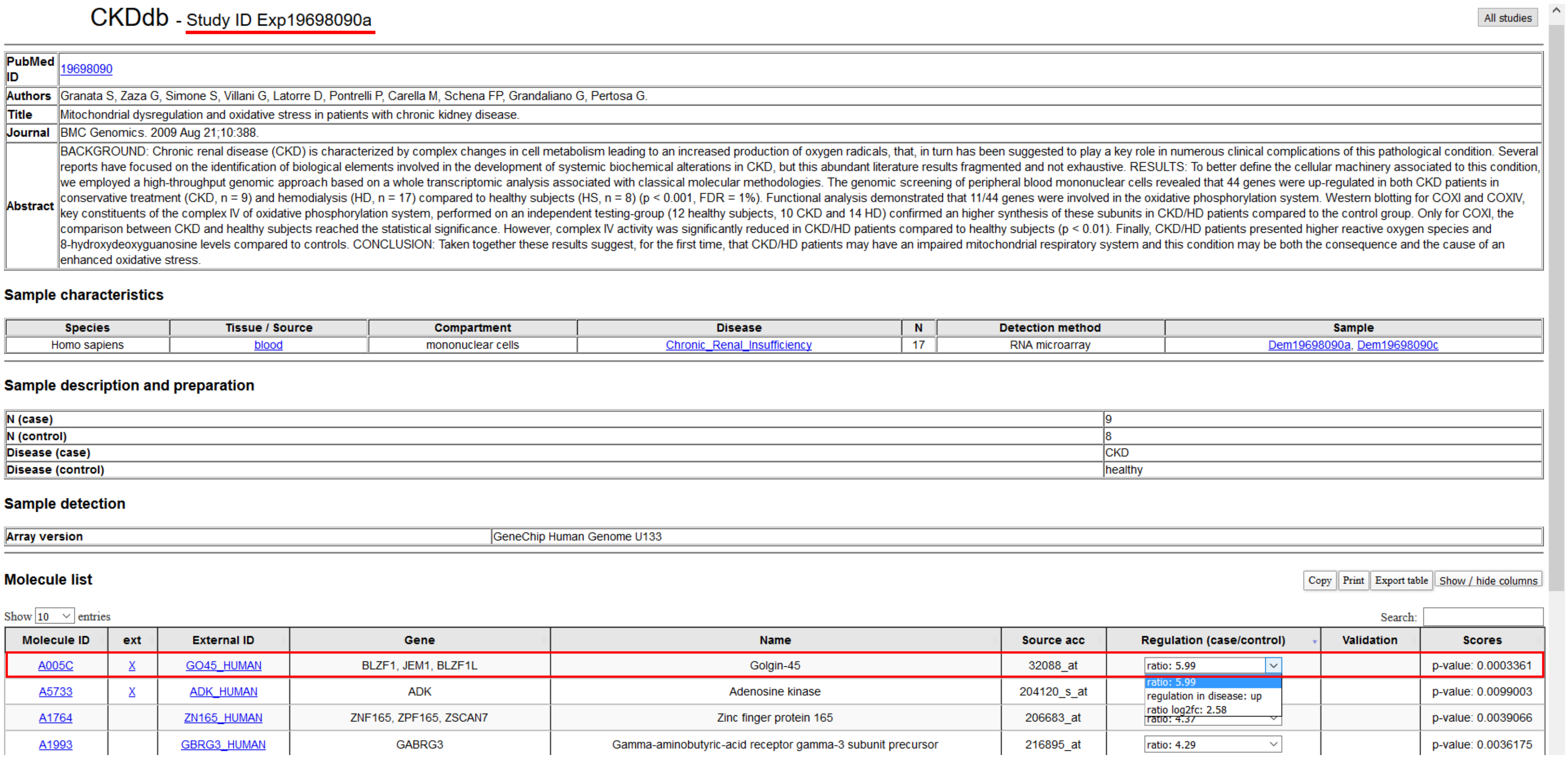

CKDdb - Study ID Exp19698090a

All studies

**PubMed ID**	19698090
**Authors**	Granata S, Zaza G, Simone S, Villani G, Latorre D, Pontrelli P, Carella M, Schena FP, Grandaliano G, Pertosa G.
**Title**	Mitochondrial dysregulation and oxidative stress in patients with chronic kidney disease.
**Journal**	BMC Genomics. 2009 Aug 21;10:388.
**Abstract**	BACKGROUND: Chronic renal disease (CKD) is characterized by complex changes in cell metabolism leading to an increased production of oxygen radicals, that, in turn has been suggested to play a key role in numerous clinical complications of this pathological condition. Several reports have focused on the identification of biological elements involved in the development of systemic biochemical alterations in CKD, but this abundant literature results fragmented and not exhaustive. RESULTS: To better define the cellular machinery associated to this condition, we employed a high-throughput genomic approach based on a whole transcriptomic analysis associated with classical molecular methodologies. The genomic screening of peripheral blood mononuclear cells revealed that 44 genes were up-regulated in both CKD patients in conservative treatment (CKD, n = 9) and hemodialysis (HD, n = 17) compared to healthy subjects (HS, n = 8) (p < 0.001, FDR = 1%). Functional analysis demonstrated that 11/44 genes were involved in the oxidative phosphorylation system. Western blotting for COXI and COXIV, key constituents of the complex IV of oxidative phosphorylation system, performed on an independent testing-group (12 healthy subjects, 10 CKD and 14 HD) confirmed an higher synthesis of these subunits in CKD/HD patients compared to the control group. Only for COXI, the comparison between CKD and healthy subjects reached the statistical significance. However, complex IV activity was significantly reduced in CKD/HD patients compared to healthy subjects (p < 0.01). Finally, CKD/HD patients presented higher reactive oxygen species and 8-hydroxydeoxyguanosine levels compared to controls. CONCLUSION: Taken together these results suggest, for the first time, that CKD/HD patients may have an impaired mitochondrial respiratory system and this condition may be both the consequence and the cause of an enhanced oxidative stress.

Sample characteristics

Species	Tissue / Source	Compartment	Disease	N	Detection method	Sample
Homo sapiens	blood	mononuclear cells	Chronic_Renal_Insufficiency	17	RNA microarray	Dem19698090a, Dem19698090c

Sample description and preparation

**N (case)**	9
**N (control)**	8
**Disease (case)**	CKD
**Disease (control)**	healthy

Sample detection

**Array version**	GeneChip Human Genome U133

Molecule list

Copy | Print | Export table | Show / hide columns

Show 10 entries

Search:

Molecule ID	ext	External ID	Gene	Name	Source acc	Regulation (case/control)	Validation	Scores
A005C	X	GO45_HUMAN	BLZF1, JEM1, BLZF1L	Golgin-45	32088_at	ratio: 5.99		p-value: 0.0003361
A5733	X	ADK_HUMAN	ADK	Adenosine kinase	204120_s_at	ratio: 5.99 / regulation in disease: up / ratio log2fc: 2.58		p-value: 0.0099003
A1764		ZN165_HUMAN	ZNF165, ZPF165, ZSCAN7	Zinc finger protein 165	206683_at	ratio: 4.37		p-value: 0.0039066
A1993		GBRG3_HUMAN	GABRG3	Gamma-aminobutyric-acid receptor gamma-3 subunit precursor	216895_at	ratio: 4.29		p-value: 0.0036175

Figure S3 Structure of the CKDdb at the study page view. First headers enclose the main bibliometric data associated with a research article. The second and third headers are with respect to the description of the sample itself and several steps in their processing till reach molecule identification. The fourth header corresponds to the incoming molecules from the previous step. It lists all the molecules identified in the study with the associated statistical scores, fold-change and regulation.

CKDdb - Molecule ID A005C

Extended info | All molecules

**gene**	BLZF1, JEM1, BLZF1L
**name**	Golgin-45
**species**	Homo sapiens

Molecule reference

SwissProt	UniProt	EnsEMBL	GeneID	UniGene	OMIM
GO45_HUMAN	Q9H2G9	ENSG00000117475	8548	Hs.130746	608692

Functions and classifications

**GO**	C:Golgi lumen, C:Golgi membrane, C:nucleus, F:DNA binding, F:sequence-specific DNA binding transcription factor activity, P:cell proliferation, P:Golgi organization, P:Golgi to plasma membrane protein transport, P:mitotic cell cycle, P:regulation of cell growth, P:regulation of transcription from RNA polymerase II promoter, C:cytoplasm, C:Golgi apparatus
**UniProt**	ADP-ribosylation, Alternative splicing, Coiled coil, Complete proteome, Cytoplasm, ER-Golgi transport, Golgi apparatus, Nucleus, Phosphoprotein, Polymorphism, Protein transport, Reference proteome, Transport, Ubl conjugation
**PADB**	transport, storage, endocytosis, exocytosis, vesicles

Studies, tissues and diseases

Study ID	Species	N	Tissue / Source	Compartment	Disease	Biomarker	Fold change in disease	P-value	Detection	Mol. modification	Validation	PubMed/DOI
Exp19698090a	Homo sapiens	17	blood	mononuclear cells	Chronic_Renal_Insufficiency (Chronic kidney disease)		5.99	0.0003361	RNA microarray			19698090
Exp19698090b	Homo sapiens	25	blood	mononuclear cells	Dialysis (hemodialysis)		2.39	0.0172	RNA microarray			19698090
Exp19698090b	Homo sapiens	25	blood	mononuclear cells	Dialysis (hemodialysis)		2.55	0.0244	RNA microarray			19698090
Exp20485333	Homo sapiens	20	blood	whole	Glomerulonephritis (IgA nephropathy)		0.27	0.003048	RNA microarray			20485333
Exp21752957b	Homo sapiens	22	kidney	tubuli	Diabetes (diabetic kidney disease)		0.34	< 0.05	RNA microarray			21752957
Exp23505438a	Homo sapiens		kidney	proximal tubular cells	Diabetes (diabetic nephropathy)		0.54	0.001	cDNA microarray			23505438
Exp23505438b	Homo sapiens		kidney	proximal tubular cells	Diabetes (diabetic nephropathy)		0.53	0.012	cDNA microarray			23505438
Exp24172336	Mus musculus	6	kidney	cortex	AKI (AKI)		1.28	0.019889449	LC-MS/MS			24172336
Exp26317775a	Homo sapiens	53	kidney	biopsy specimens	Chronic_Renal_Insufficiency (CKD)		0.45	1.22E-04	RNA microarray			26317775

Figure S4 View of the of a specific molecule entry in CKDdb. In the first header is displayed the linkage to external databases. Following it we have represented the associated function and classification and as well our own type of tag for functional classification (derived from PADB). The third header corresponds to the number of occurrences of the molecule across all the studies, disease conditions, species, and tissue/fluid source.

CKDdb - Sample ID Dem19698090a

**IDs**	**Sample**	Dem19698090a
	**Study**	Exp19698090a
**General**	**Species**	Homo sapiens
	**Cohort size N**	9
	**Disease status**	CKD
	**Age [years]**	49.12 +/- 9.82
	**Gender**	5/4 (M/F)
**Clinical**		
	**BMI**	22.1 +/- 0.92
	**Systolic BP [mmHg]**	135 +/- 17.4
	**Diastolic BP [mmHg]**	83.11 +/- 6.23
	**U-albumin [g/l]**	4.1 +/- 0.24 (g/dl)
	**GFR (Cockcroft-Gault equation)**	41.4 +/- 4.3 ml/min/1.73 m2
	**CKD stage**	stage 2-3
**Sample**	**Tissue type**	blood

Figure S5 Clinical data (demographics page view) is also represented in the CKDdb database and is linked with the study page and vice-versa.

After data thresholding (Pvalue: <0.05 and Fold-change: down-regulated ≤0.66 and up-regulated ≥1.5) we used the functionality tag clustering system of the PADB database to classify total, up-regulated and down-regulated molecules.

Functionality tag clustering – [CKDdb]

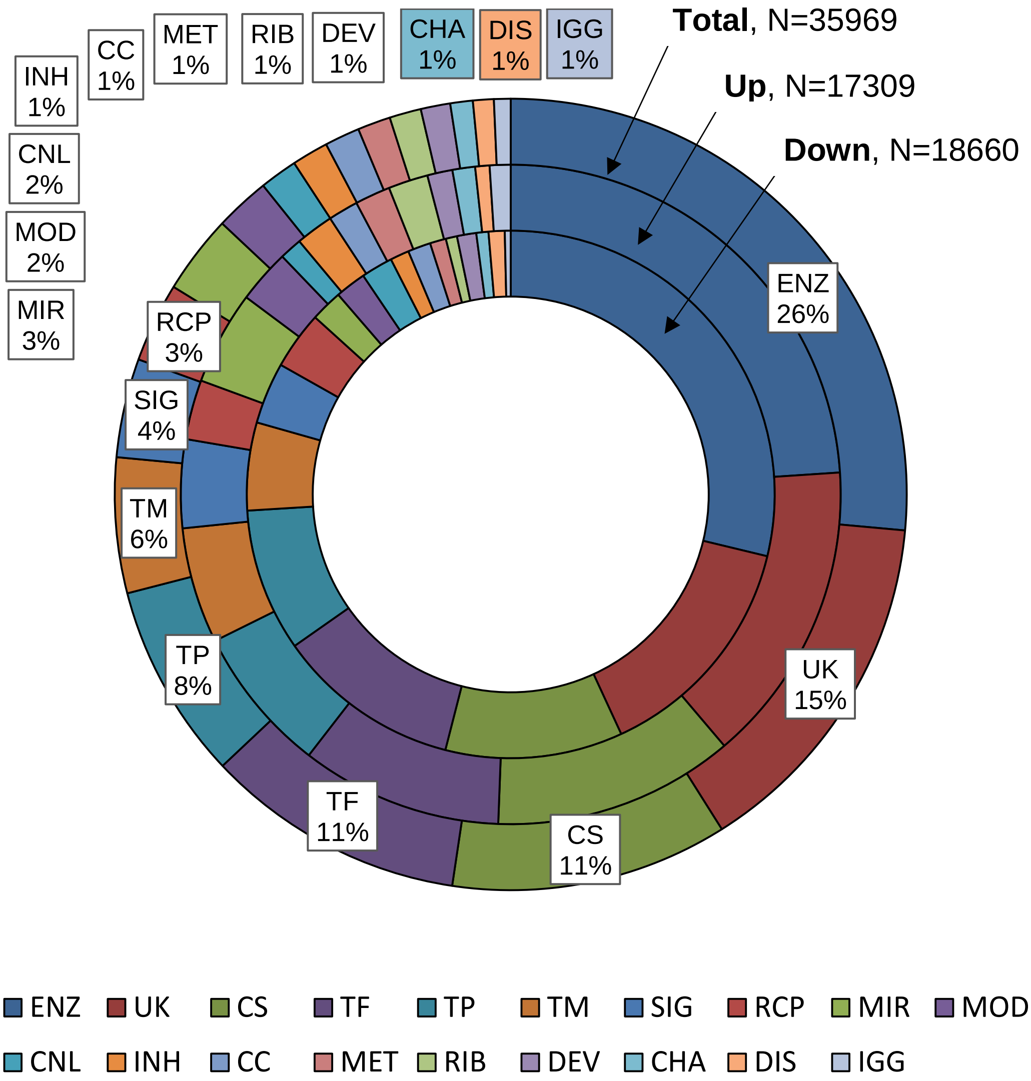

Figure S6 - Functionality tag clustering of the total differentially expressed molecules (fold-change: down-regulated ≤0.66 and up-regulated ≥1.5 and Pvalue <0.05) for the CKDdb database. ENZ: enzyme, enzymatic properties; UK: unknown; CS: cell shape (cytoskeleton, cell adhesion, morphology, cell junction, cellular structures, extracellular matrix); TF: transcription and translation, gene regulation; TP: transport, storage, endocytosis, exocytosis, vesicles; TM: transmembrane; SIG: signalling; RCP: receptor; MIR: microRNA; MOD: modulator, regulator; CNL: channel; INH: inhibitor (protease, kinase, other enzymes, pathways); CC: cell cycle (turnover, mitosis, meiosis); MET: metabolite; RIB: ribosome; DEV: development, cell growth, differentiation, morphogenesis; CHA: chaperone, chaperonin; DIS: disease; IGG: immunoglobulin.

Term clustering – global – CKDdb database

Hereafter data thresholding (Pvalue: <0.05 and Fold-change: down-regulated ≤0.25 and up-regulated ≥4) we used the Cytoscape plugin ClueGO to identify the main associated biological processes using gene ontology (GO). Red node colour denotes an increased regulation of the biological process and green a decrease. Nodes presenting grey colour means that they have an equal number of genes/proteins up and down regulated.

We also cross-compared blood, kidney and urine data sets from mRNA and protein studies and identifying the involved pathway(s) terms from KEGG and WikiPathways, splitting it by regulation type: up against down.

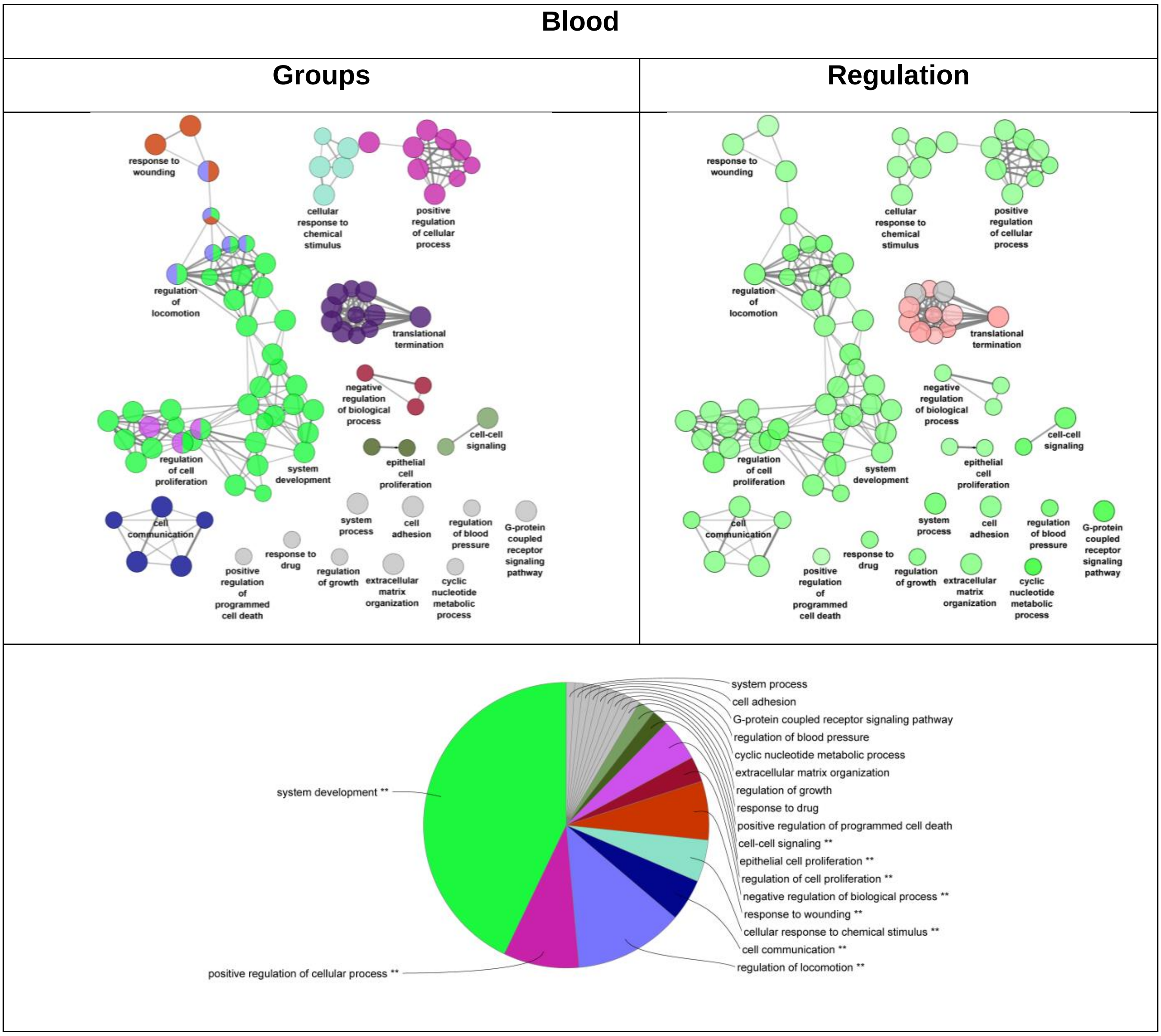

Figure S7 - GO group functionality (biological process) and molecular regulation for blood (only human data across all diseases present in the database). GO term/pathway network connectivity (kappa score): 0.4; GO term fusion; pathways/term pV≤0.01. Data thresholding, Pvalue: <0.05 and Fold-change: down-regulated ≤0.25 (green nodes) and up-regulated ≥4 (red nodes). The pie-chart represents cluster#1 – down-regulated with their associated GO terms.

Kidney	
Groups	Regulation

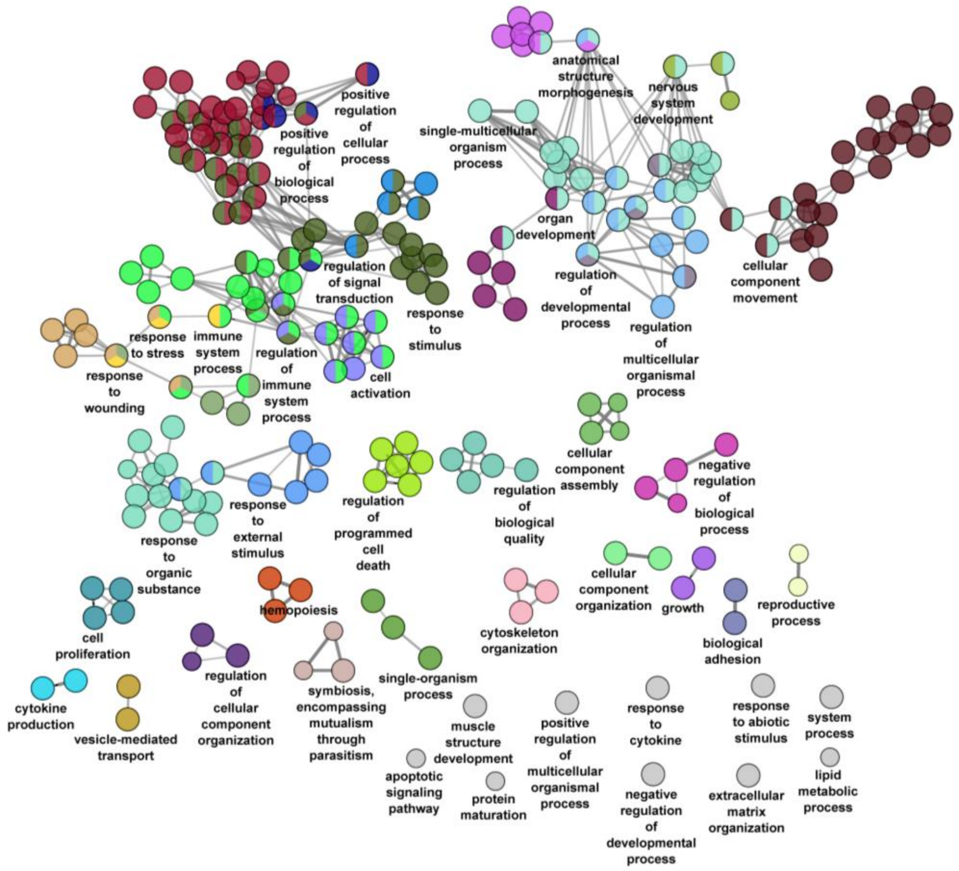

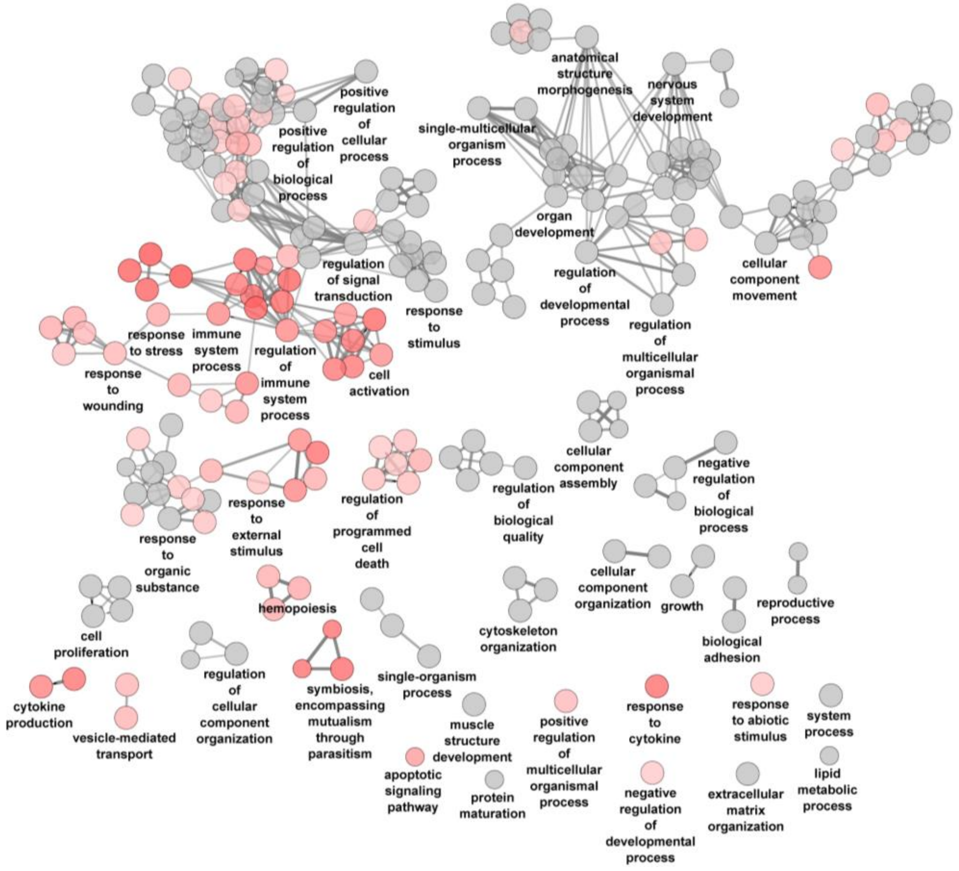

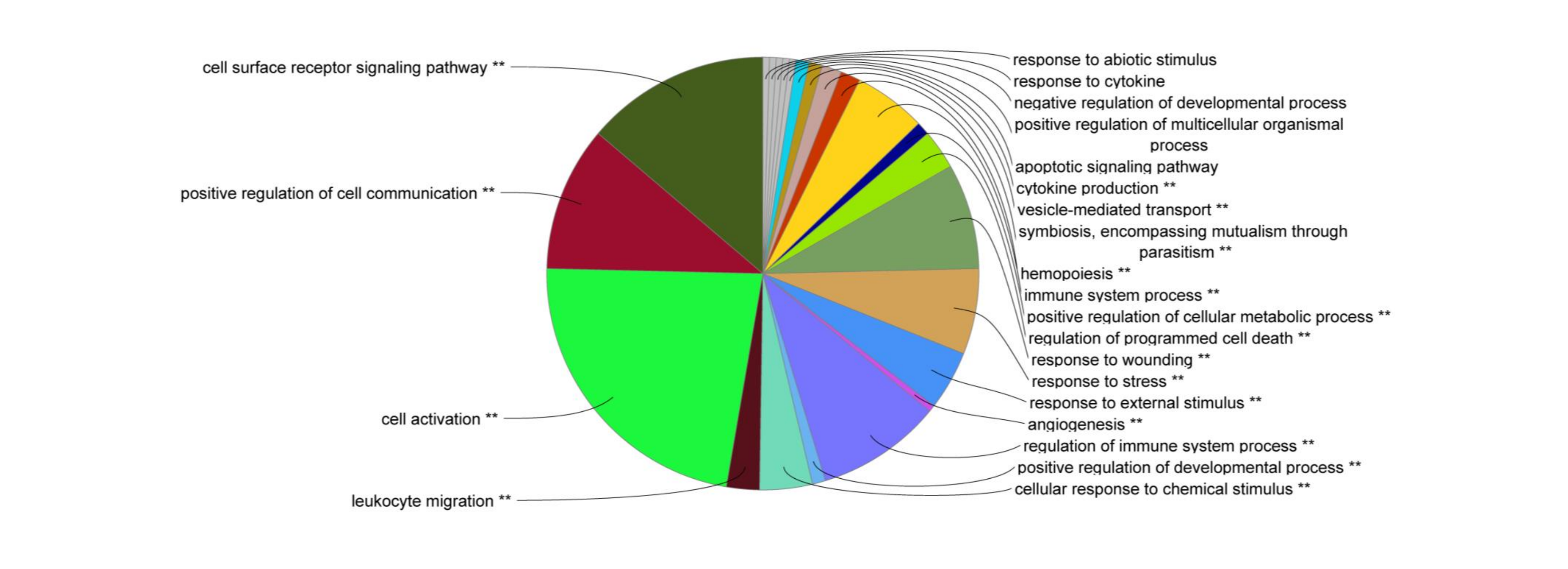

Figure S8 - GO group functionality (biological process) and molecular regulation for the kidney tissue (only human data across all diseases present in the database). GO term/pathway network connectivity pathway network connectivity (kappa score): 0.4; GO term fusion; pathways/term pV≤0.01. Data thresholding, Pvalue: <0.05 and Fold-change: down-regulated ≤0.25 (green nodes) and up-regulated ≥4 (red nodes). The pie-chart represents cluster#2 – up-regulated with their associated GO terms.

Urine	
**Groups**	**Regulation**

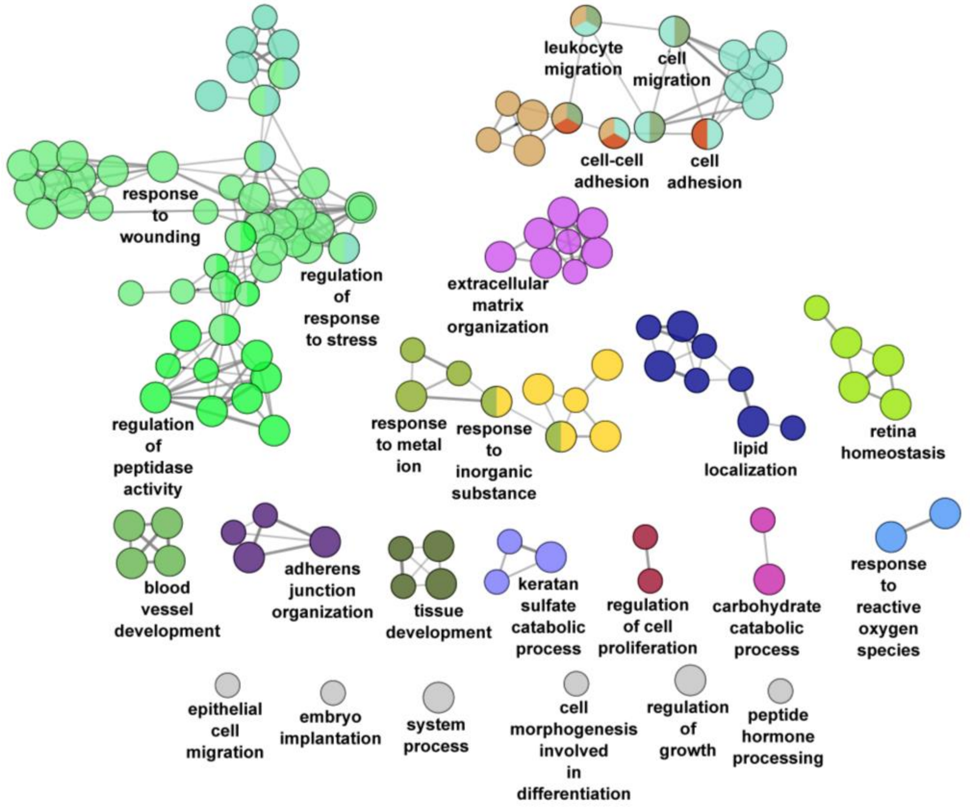

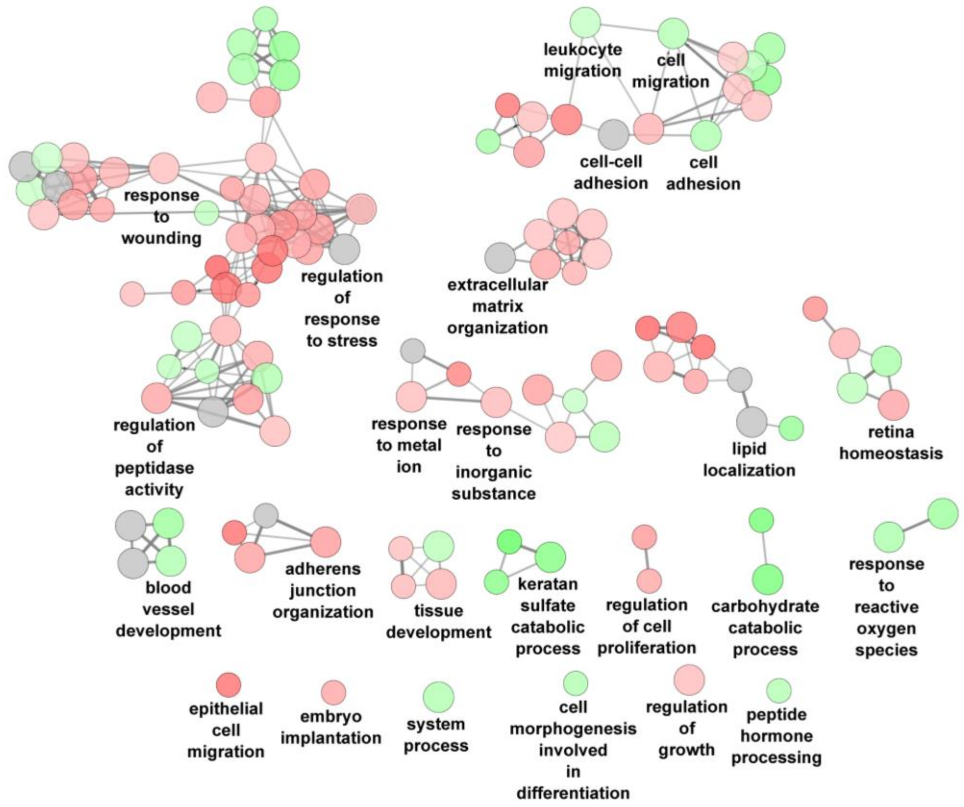

Cluster#1

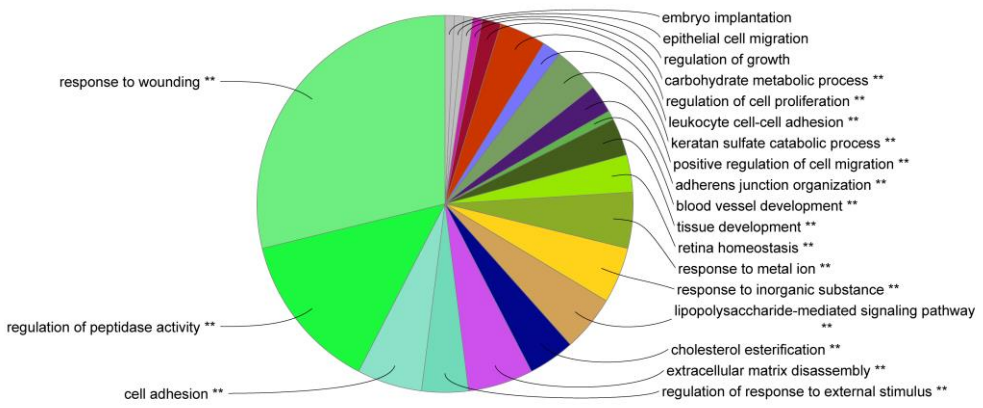

Cluster#2

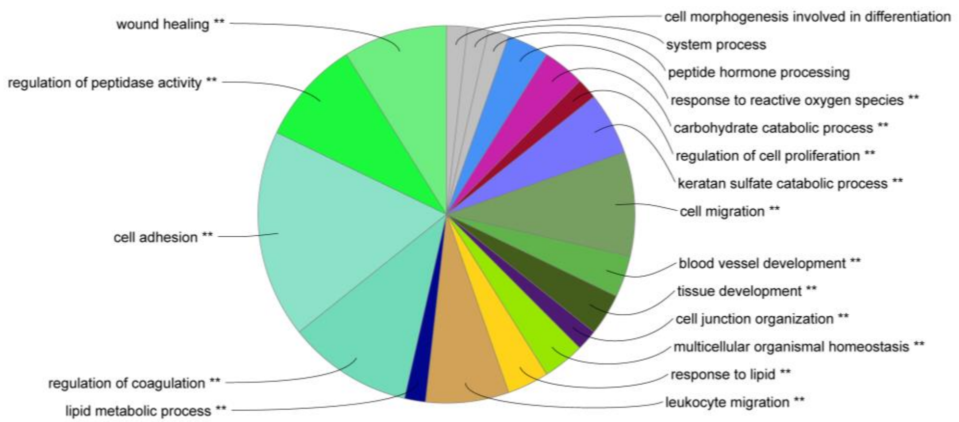

Figure S9 - GO group functionality (biological process) and molecular regulation for urine (only human data across all diseases present in the database). GO term/pathway network connectivity pathway network connectivity (kappa score): 0.4; GO term fusion; pathways/term pV≤0.01. Data thresholding, Pvalue: <0.05 and Fold-change: down-regulated ≤0.25 (green nodes) and up-regulated ≥4 (red nodes). The pie-chart represents respectively cluster#1 – down-regulated and cluster#2 – up-regulated with their associated GO terms.

Term clustering – pathways (KEGG and WikiPathways)

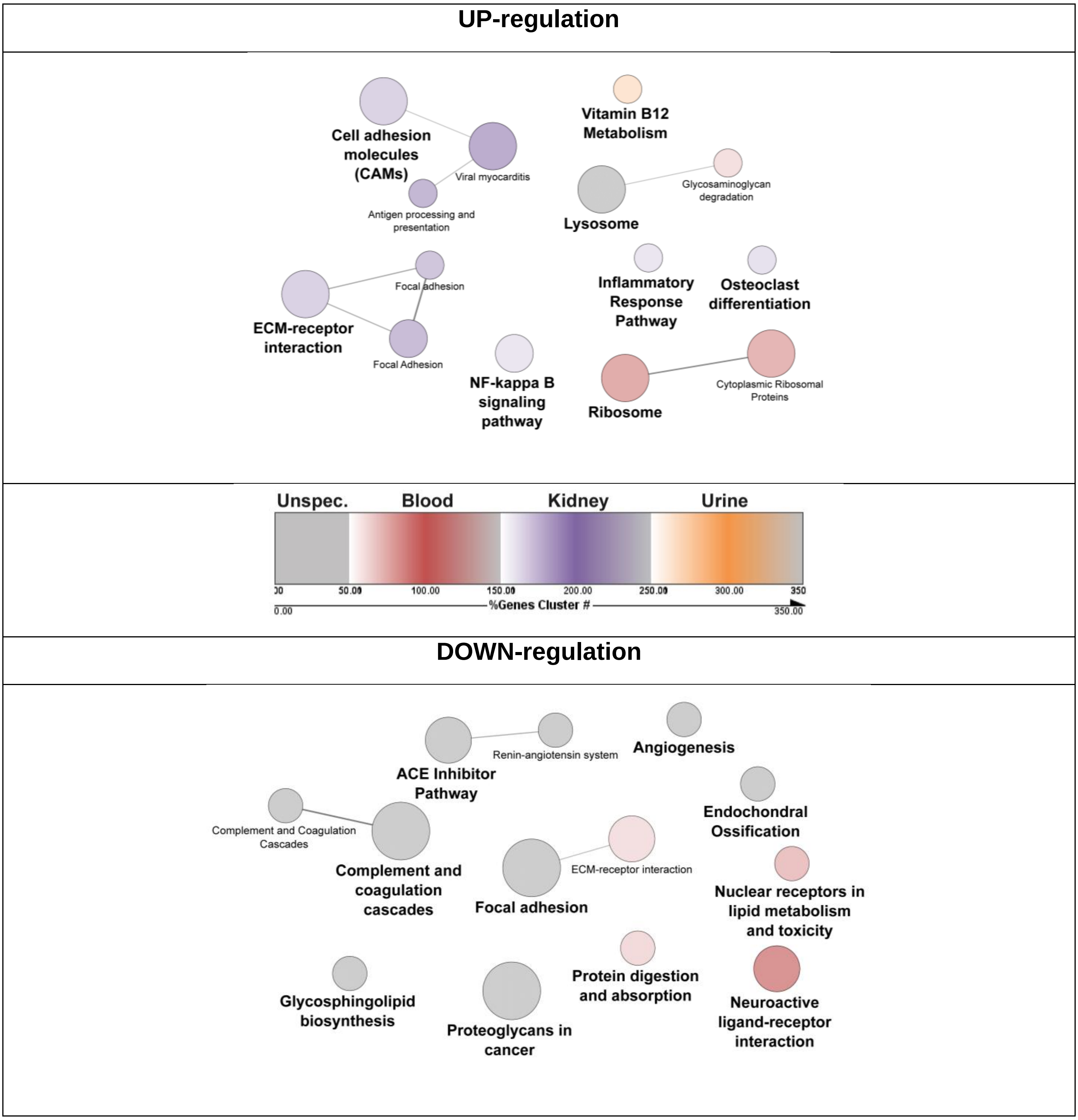

Figure S10 - ClueGO analysis of the associated pathway(s) terms from KEGG and WikiPathways. Cross-comparison between kidney, blood and urine datasets.

Targeted analysis: case study with a subset of the CKDdb database - CRI datasets

Table S1 Dataspace description of the 19 studies used in our analysis. EXPREF: is the PMID identifier preceded by "Exp". NA: no mention within the study at least on a straightforward manner. Type: states the molecules associated in the study; G: gene, M: metabolite; MIR: miRNA; P: protein. Only human studies were used in this analysis.

EXPREF	total	N (case)	N (control)	disease case	disease control	source	detection method	Type
Exp19698090a	17	9	8	CKD	healthy	blood	RNA microarray	G
Exp23809614a	83	63	20	End-stage renal failure	NA	blood	RNA microarray	
Exp23935909	24	14	10	CKD	healthy	blood	RNA microarray	
Exp24189015b	14	9	5	CKD stage 3-4	healthy	blood	RNA microarray	
Exp26317775a	53	48	5	CKD	healthy	kidney	RNA microarray	
Exp20613759	41	NA	NA	Chronic kidney failure	NA	blood	CE-TOF MS	M
Exp23220422b	10	NA	NA	CKD_stage_4	CKD stage 2	blood	GC/MS, LC/MS	
Exp24048377a	30	15	15	CKD	healthy	urine	2D-NMR	
Exp24429397	80	40	40	End-stage renal disease	stable	blood	LC-ESI-MS/MS	
Exp26266360	38	19	19	CKD	NA	blood	UPLC-Q-TOF/MS	
Exp21891774a	75	53	22	CKD_stage_4	healthy	blood	RT-qPCR	MIR
Exp21891774b	22	13	9	CKD_stage_4	healthy	urine	RT-qPCR	
Exp23946286	39	32	7	CKD	healthy	urine	qRT-PCR	
Exp24184689	10	NA	NA	CKD	healthy	blood	miRNA microarray	
Exp17095863	10	7	3	CKD	healthy	urine	Antibody Array	P
Exp21183621	24	17	7	CKD	healthy	blood	ProtoArray	
Exp25622820a	60	30	30	CKD stage 1-2	healthy	blood	MALDI-TOF/TOF	
Exp25622820b	60	30	30	CKD stage 3-4	healthy	blood	MALDI-TOF/TOF	
Exp25622820c	60	30	30	CKD stage 5	healthy	blood	MALDI-TOF/TOF	

Functionality tag clustering – [focused dataset]

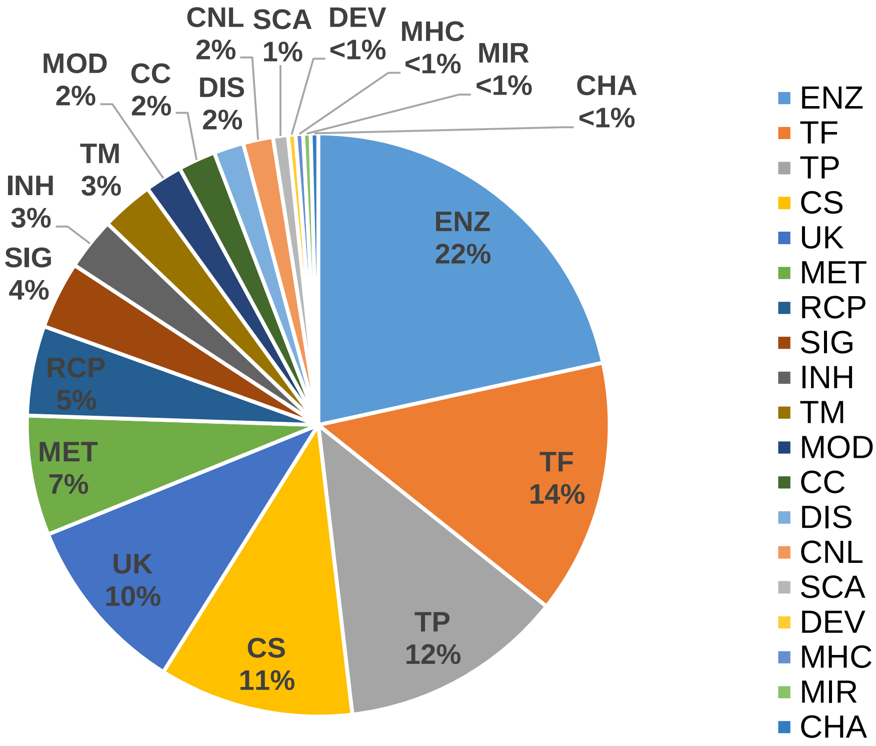

Figure S11 - Functionality tag clustering of the total differentially expressed molecules (N=241, fold-change <0.77 & >1.3 and Pvalue <0.05) for the focused dataset. ENZ: enzyme, enzymatic properties; TF: transcription and translation, gene regulation; TP: transport, storage, endocytosis, exocytosis, vesicles; CS: Cell shape (cytoskeleton, cell adhesion, morphology, cell junction, cellular structures, extracellular matrix); UK: unknown; MET: metabolite; RCP: receptor; SIG: signalling; INH: inhibitor (protease, kinase, other enzymes, pathways); TM: transmembrane; MOD: modulator, regulator; CC: cell cycle (turnover, mitosis, meiosis); DIS: disease; CNL: channel; SCA: scaffolder, docking, adaptor; DEV: development, cell growth, differentiation, morphogenesis; MHC: major histocompatibility complex component/protein cluster (MHC, HLA); MIR: microRNA; CHA: chaperone, chaperonin.

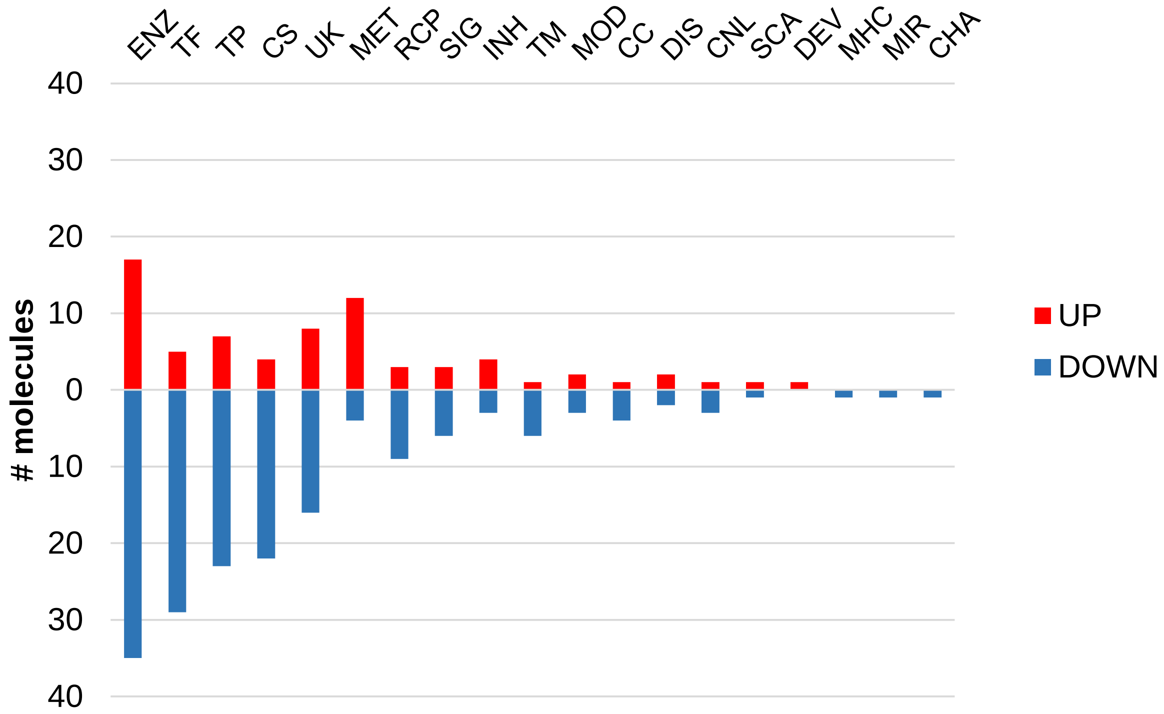

Figure S12 - Functionality tag clustering of the down (N=169) and up-regulated (N=72) molecules (fold-change ≤0.77 & >1.3 and Pvalue <0.05) for the focused dataset.

Functionality tag clustering – [unfocused dataset]

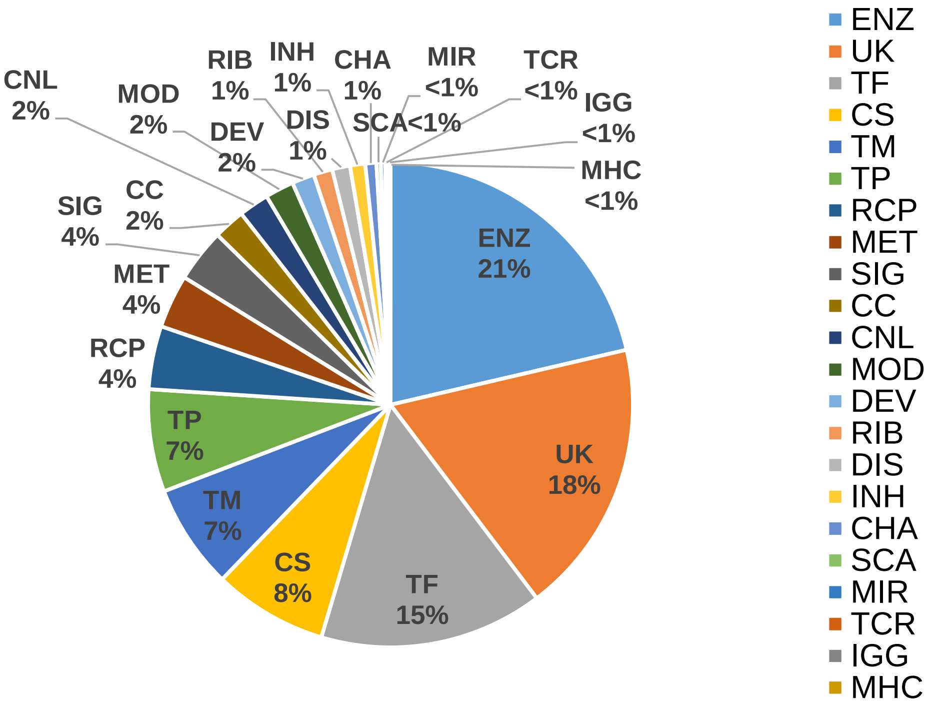

Figure S13 - Functionality tag clustering of the total differentially expressed molecules (N=4860, fold-change <0.77 & >1.3 and Pvalue <0.05) for the unfocused dataset. ENZ: enzyme, enzymatic properties; UK: unknown; TF: transcription and translation, gene regulation; CS: Cell shape (cytoskeleton, cell adhesion, morphology, cell junction, cellular structures, extracellular matrix); TM: transmembrane; TP: transport, storage, endocytosis, exocytosis, vesicles; RCP: receptor; MET: metabolite; SIG: signalling; CC: cell cycle (turnover, mitosis, meiosis); CNL: channel; MOD: modulator, regulator; DEV: development, cell growth, differentiation, morphogenesis; RIB: ribosome; DIS: disease; INH: inhibitor (protease, kinase, other enzymes, pathways); CHA: chaperone, chaperonin; SCA: scaffolder, docking, adaptor; MIR: microRNA; TCR: T-cell receptor; IGG: Immunoglobulin; MHC: major histocompatibility complex component/protein cluster (MHC, HLA).

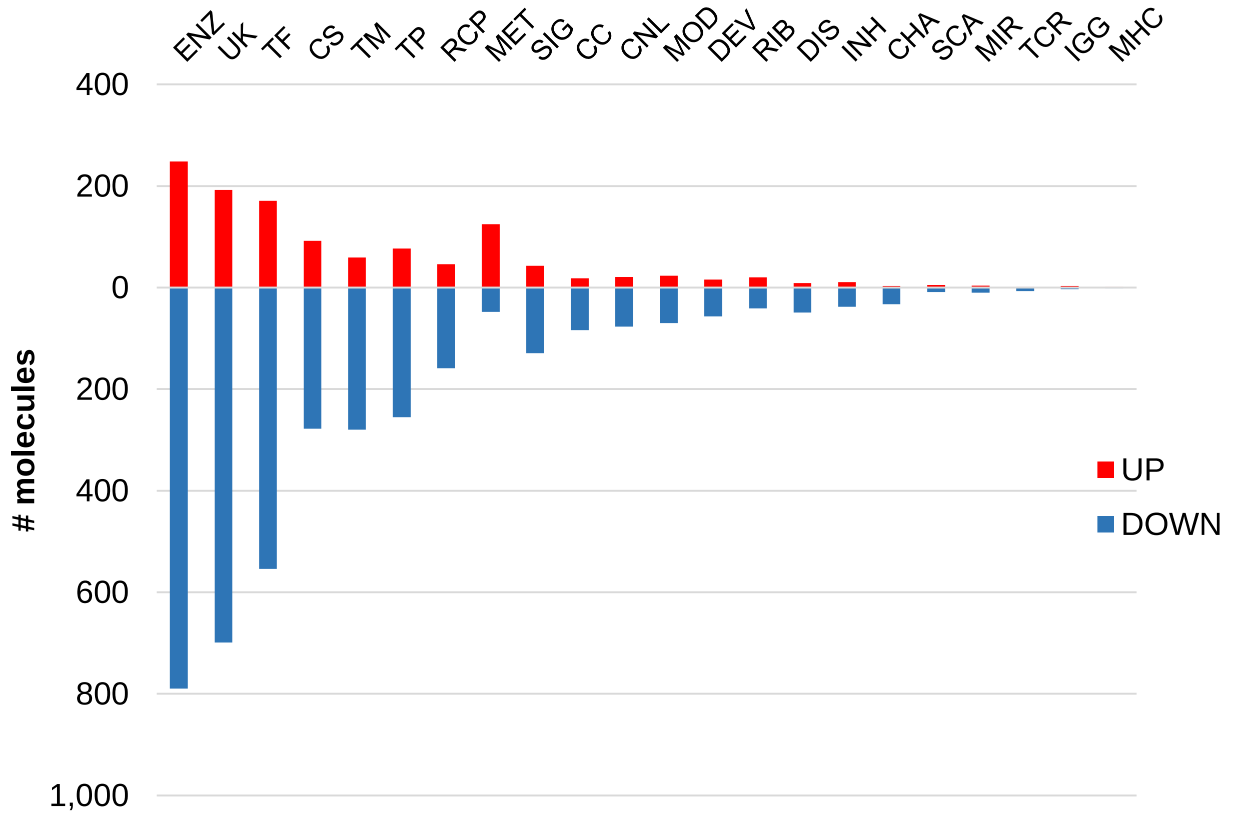

Figure S14 - Functionality tag clustering of the down (N=3672) and up-regulated (N=1188) molecules (fold-change <0.77 & >1.3 and Pvalue <0.05) for the unfocused dataset.

Gene ontology (GO) and pathway term clustering

[Focus dataset]

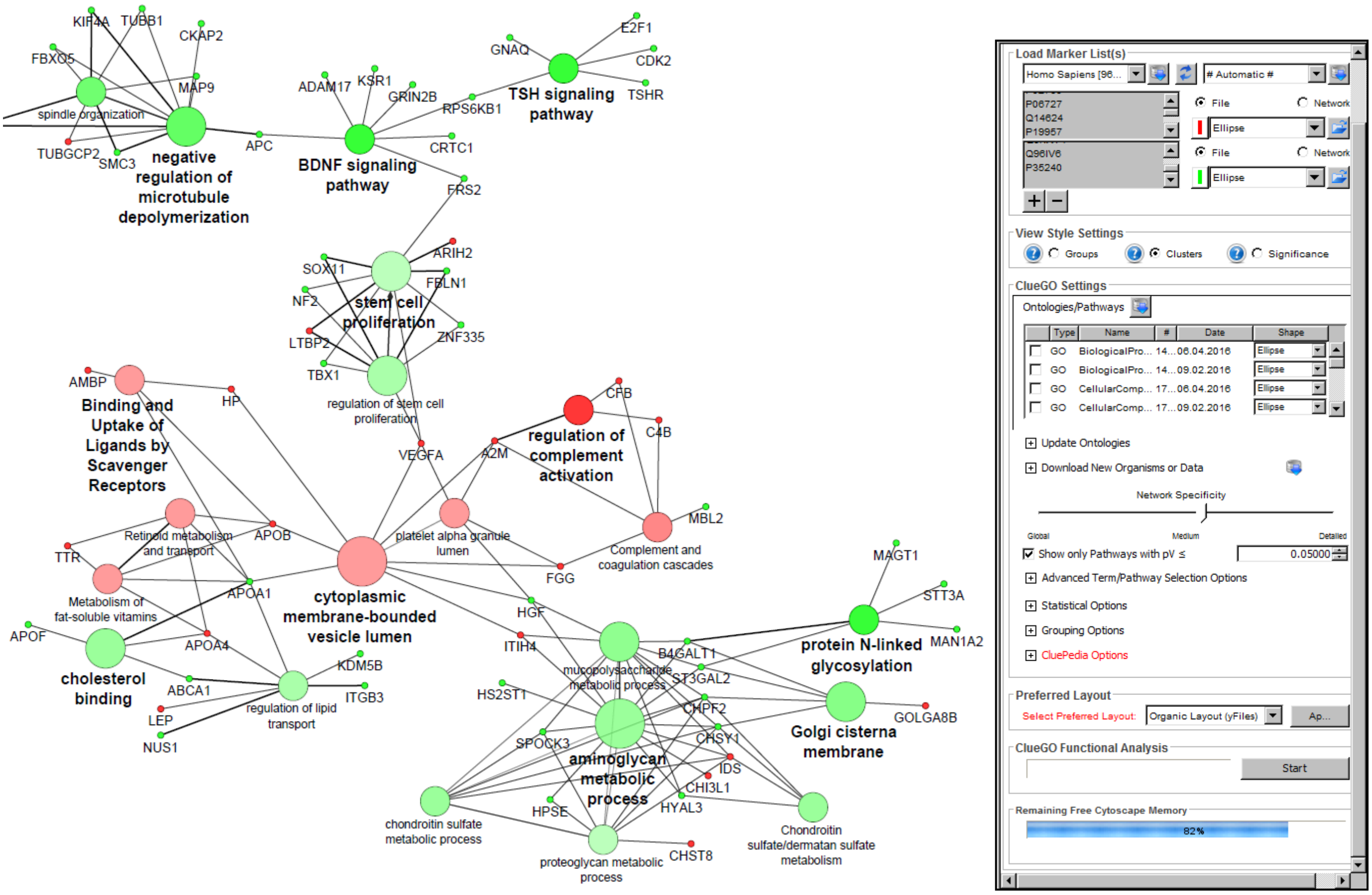

Figure S15 - Overall view of the term clustering based on molecular regulation (down: green, up: red) from GO (Biological process, immune system and molecular function) and pathway term clustering (KEGG, Reactome and WikiPathways) of the focused dataset within CRI and human data, across multi-tissues and fluid sources (blood, urine and kidney). Network merging (union) of GO terms (Biological Process, Molecular Function and Cellular component), and Pathway terms (Reactome, KEGG, and WikiPathways) into a global network performed in ClueGO.

Molecular clustering based on protein-protein interactions (PPI's)*:* GeneMania | [both datasets]

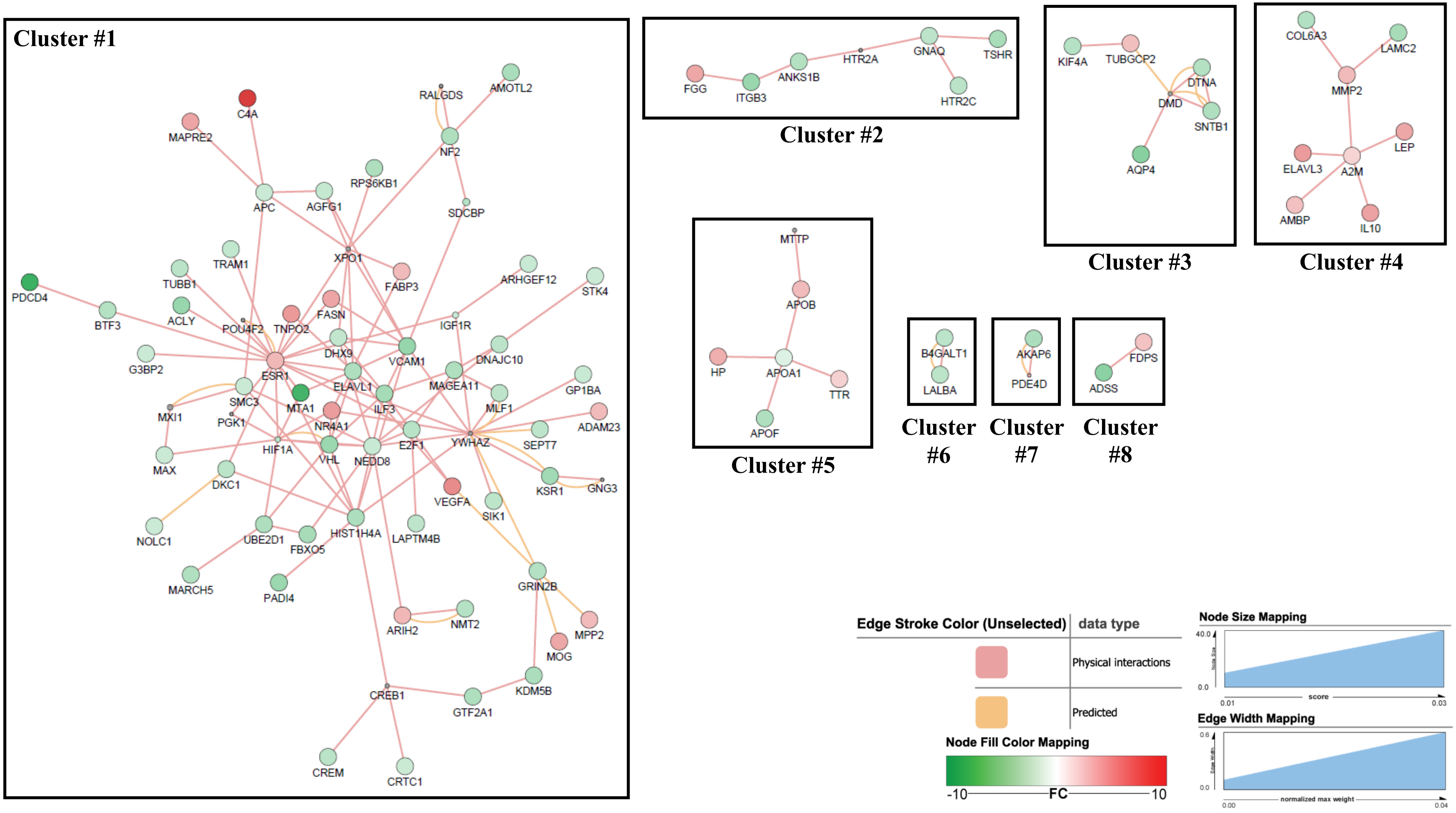

Figure S16 - Molecular clustering based on protein-protein interactions (PPI's) and regulation using both datasets (focused + unfocused) plus enrichment (grey nodes) in GeneMania.

Molecular clustering based on protein-protein interactions (PPI's)*:* GeneMania | [Focus dataset] | molecules from the BDNF and TSH signalling pathways

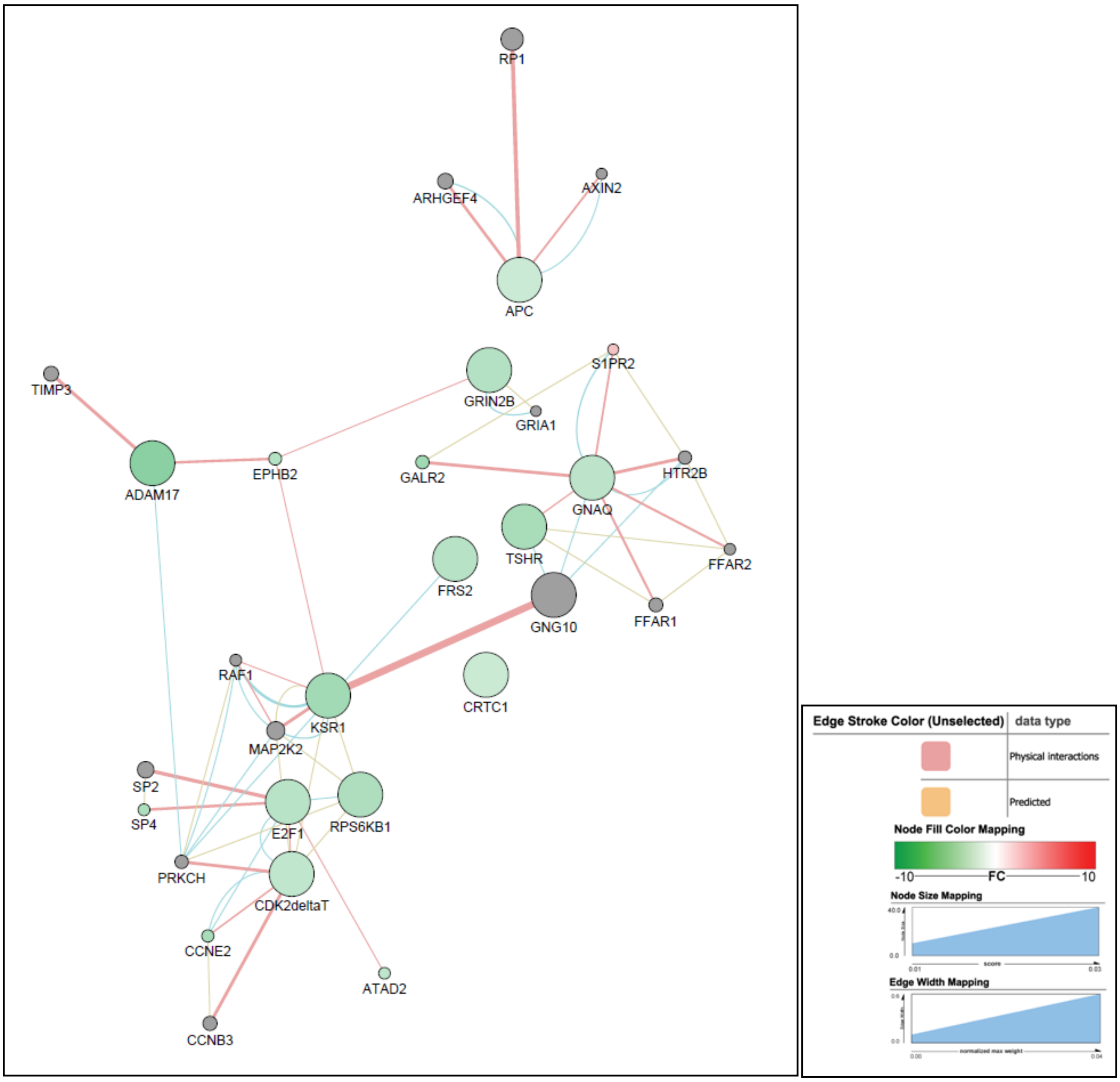

Figure S17 - Molecular clustering based on protein-protein interactions (PPI's) and regulation using the focused dataset and molecules from the BDNF and TSH signalling pathways plus enrichment (grey nodes) in GeneMania. The blue edges denote associated nodes from the same pathway.

Regulatory networks: microRNAs-target genes

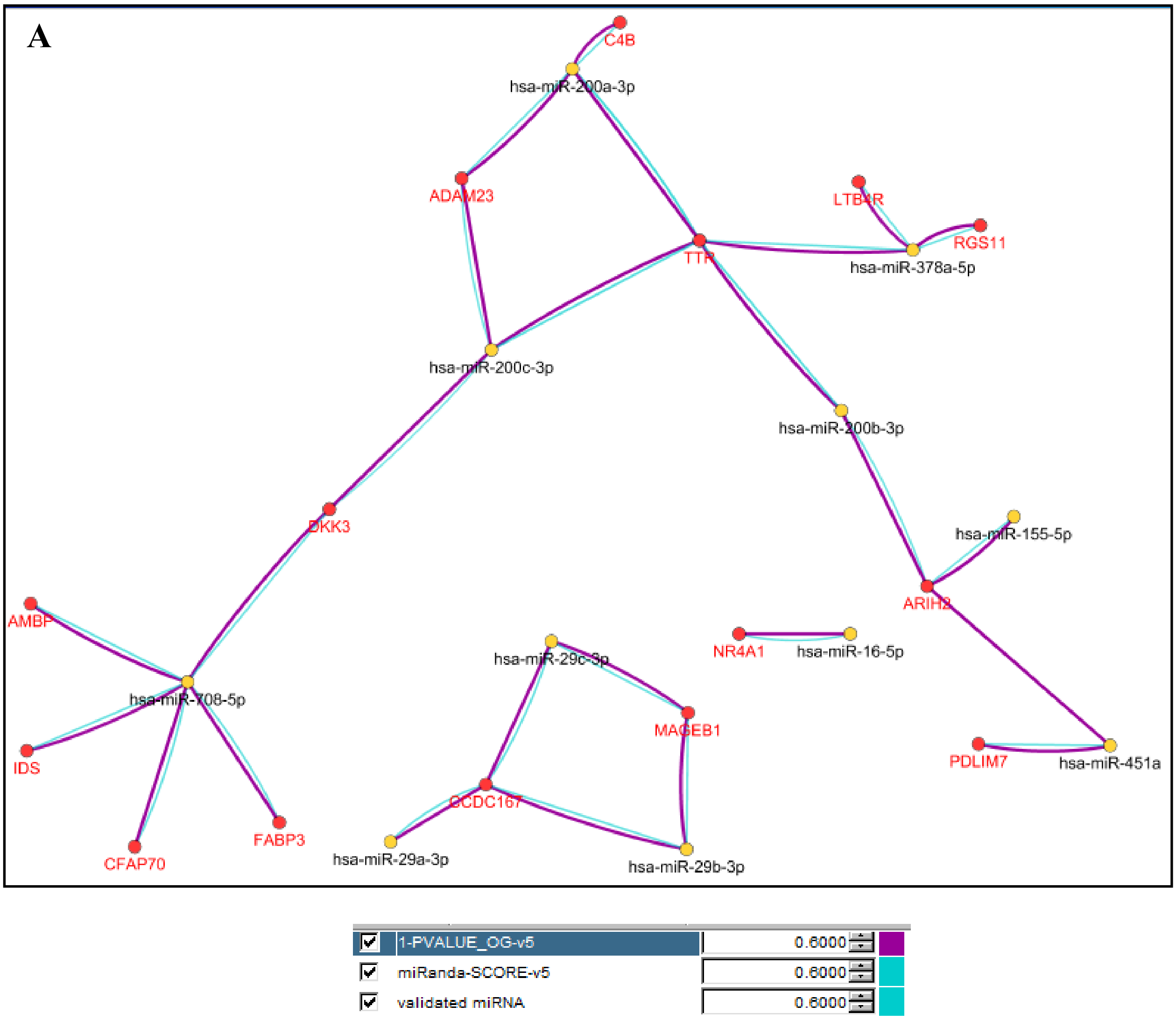

KEGG

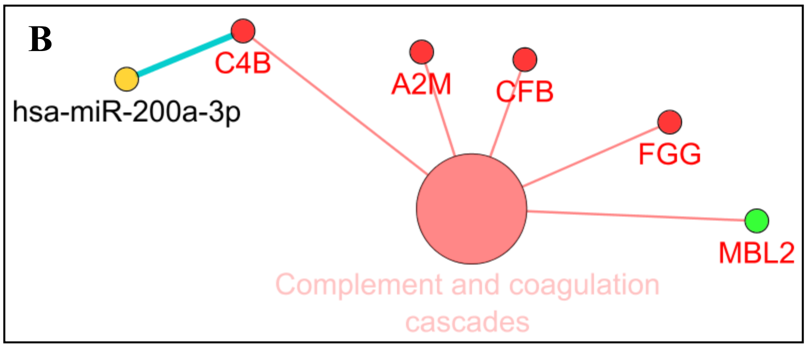

Reactome

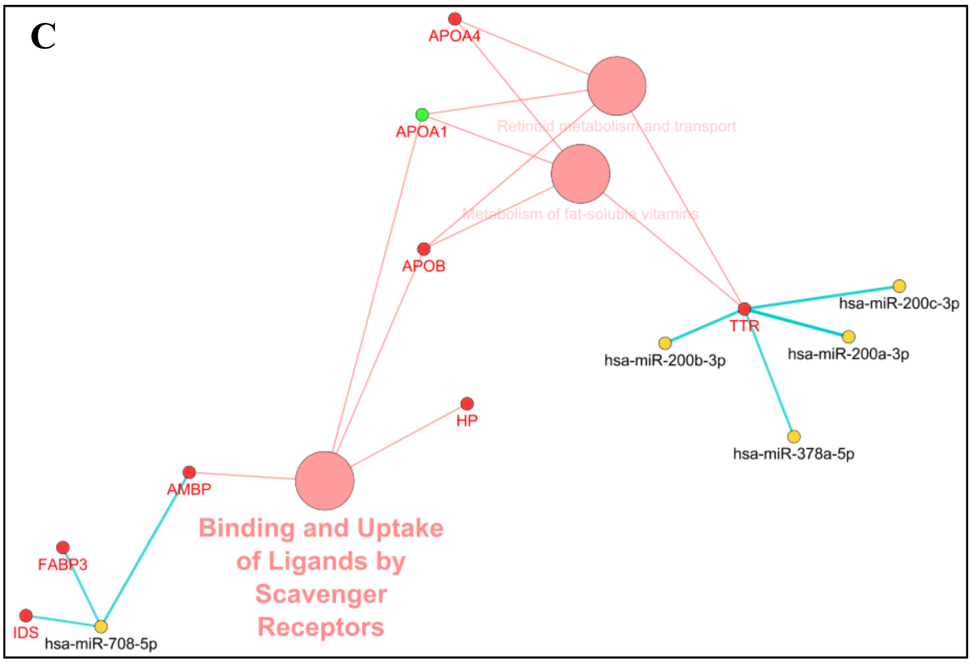

Figure S18 - Association of down-regulated microRNAs and their up-regulated gene targets (A) using miRNAs from both datasets and gene/proteins from the focused dataset via Cytoscape software and ClueGO+CluePedia application. Linkage of miRNAs to targets and their associated pathways terms B and C.

Interactome matching: gene -metabolite| [Focus dataset]

Proteoglycan biosynthesis

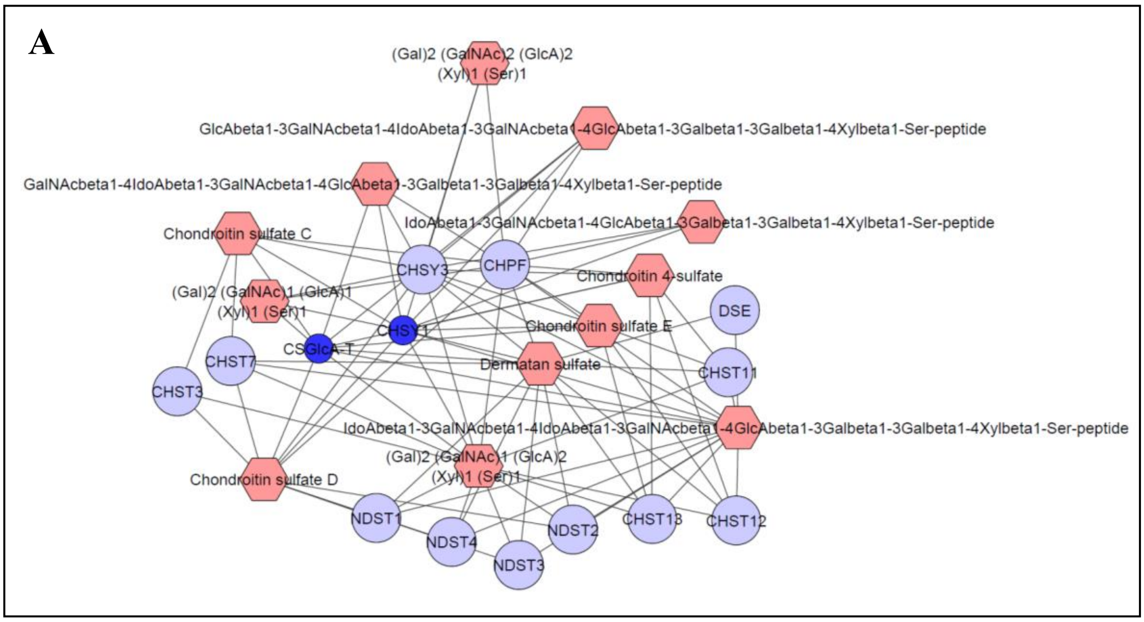

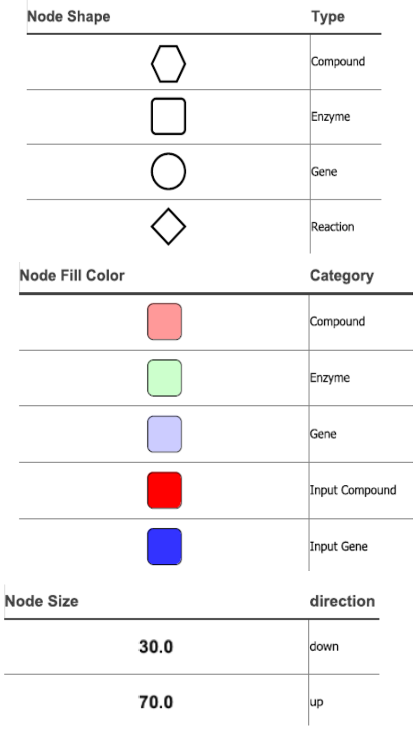

O- and *N*-glycan biosynthesis

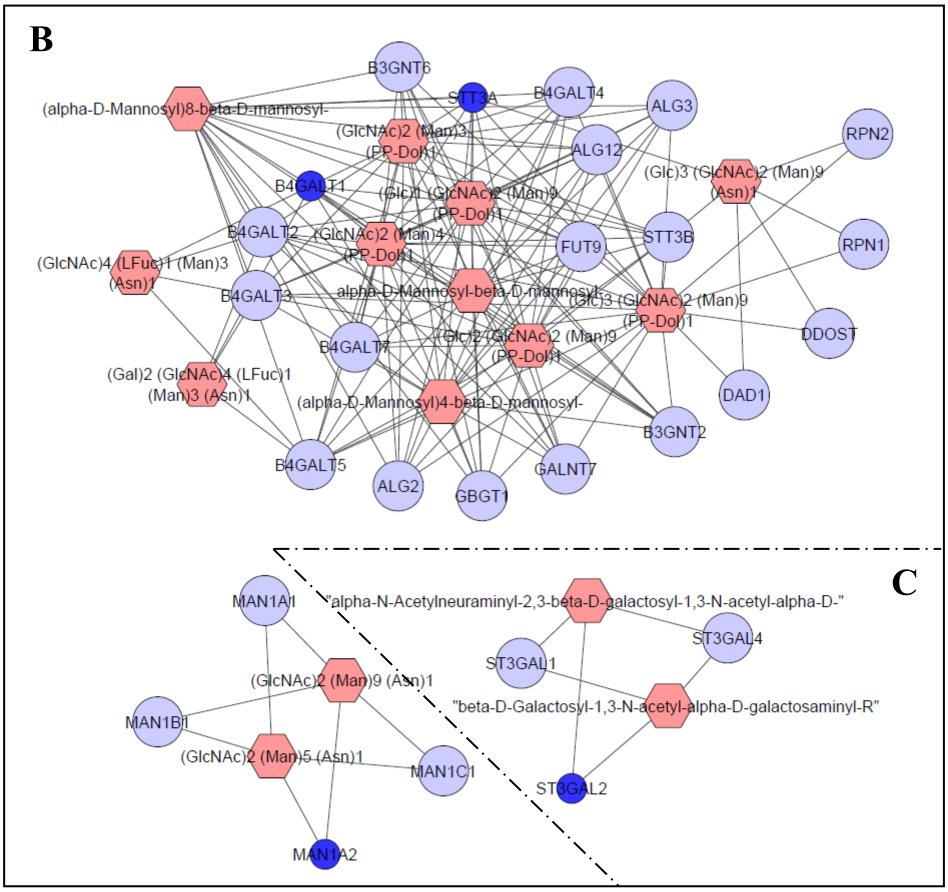

Figure S19 - Association of genes/proteins and metabolites for the Proteoglycan biosynthesis (A), N-glycan biosynthesis (B) and O-glycan biosynthesis (C) using our focused dataset via Cytoscape software and Metscape application.

Figure S20 Mapping of the molecular features into existing pathway maps

(next page)

Title: CRI pathways merged
Availability: CC BY 2.0
Last modified: 09/07/2016
Organism: Homo sapiens

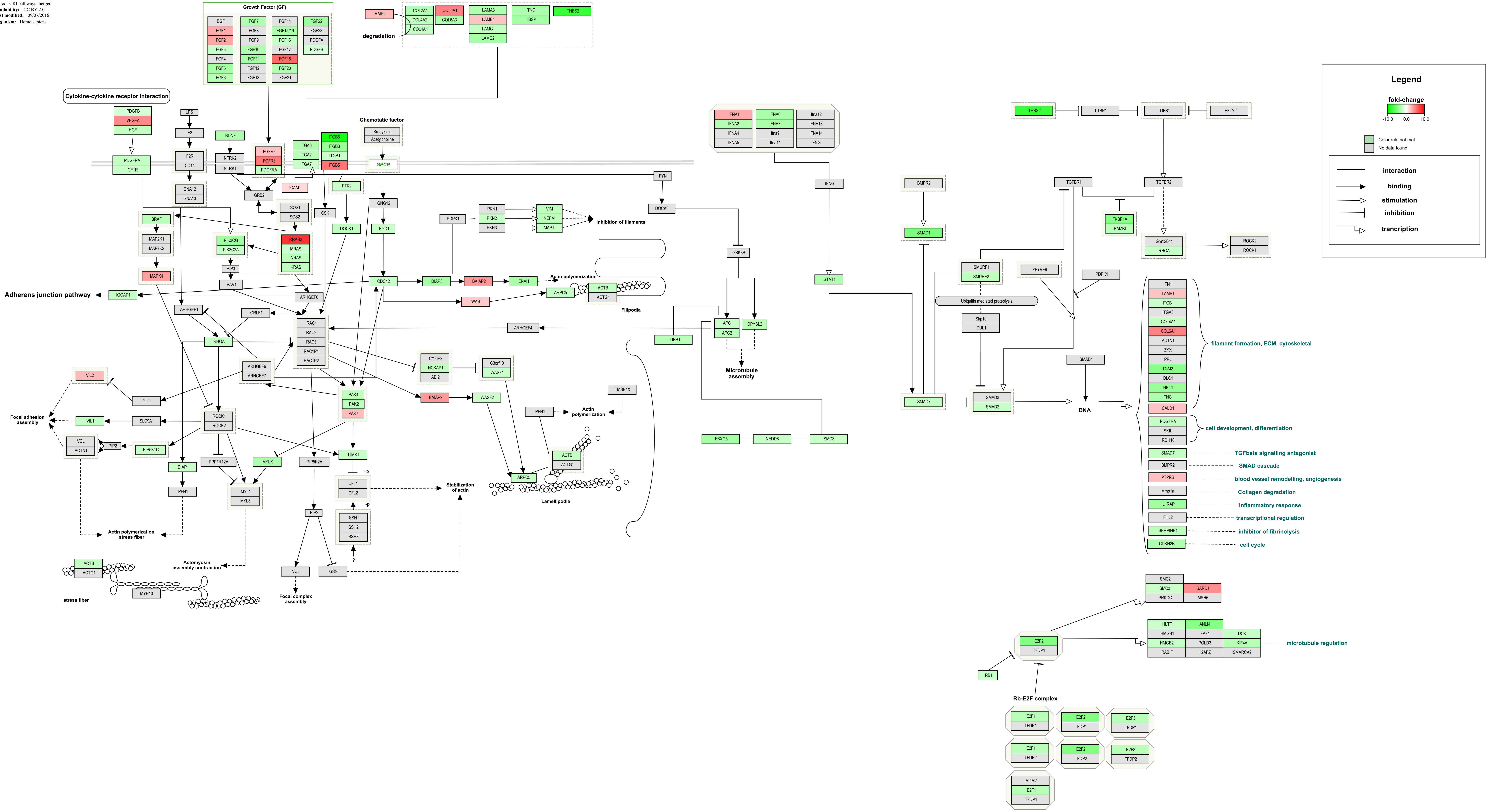

Supplement: Supplementary Information [file srep40367-s1.pdf]
